# Highly Efficient and achromatic mid-infrared silicon nitride meta-lenses

**DOI:** 10.1038/s41598-024-83728-2

**Published:** 2025-01-15

**Authors:** Abdullah Maher, Mohamed A. Swillam

**Affiliations:** Department of Physics, The American University in Cairo, New Cairo, 11835 Egypt

**Keywords:** Optical physics, Nanophotonics and plasmonics

## Abstract

Inverse design with topology optimization considers a promising methodology for discovering new optimized photonic structure that enables to break the limitations of the forward or the traditional design especially for the meta-structure. This work presents a high efficiency mid infra-red imaging photonics element along mid infra-red wavelengths band starts from 2 to 5 µm based on silicon nitride optimized material structures. The first two designs are broadband focusing and reflective meta-lens under very high numerical aperture condition (NA = 0.9). The two designs are modeled by inverse design with topology optimization problem with Kreisselmeier–Steinhauser (k–s) aggregation objective function, while the final design is depended on novel inverse design optimization problem with double aggregation objective function that can target bifocal points along the wavelength band producing high efficiency achromatic broadband multi-focal meta-lens under very high numerical apertures ($N{A}_{1} = 0.9, \, N{A}_{2}=0.88$).

## Introduction

Scientists and engineers always need new methods and techniques to break away from conventional or traditional design, especially when it’s related to better efficiency, lower costs and time savings. There are many methodologies to improve the performance and efficiency of the design that have emerged in the last few decades and are receiving great attention from researchers. One of these techniques is Topology Optimization (TO) or it’s called Shape Optimization^1,2^. Based on mathematical optimization methods and user-defined space related to a set of conditions and constraints, topology optimization optimizes the material layout to ultimately maximize the performance and efficiency of the design. Topology optimization is usually related to inverse design, where new shapes can be reversed engineered based on TO by simply determining a set of desired parameters and characteristics and let the optimizer algorithm generate a predicted solution. Inverse design with topology optimization has a great impact on improving the performance of photonic devices, where the configurations of photonic structures, whose geometry parameters are found by trial and error, where it’s derived from optimization that have a major impact on performance^3–7^. Consequently, the anticipated structure’s functionality may be significantly limited. In recent years, inverse design methodologies have been extensively utilized in the development of cutting-edge photonic structures, including photonic crystals, metamaterials, meta-surfaces, and meta-structures^8,9^, which demonstrate superior performance compared to structures constructed through empirical methods in numerous applications. In view of the findings of previous research^10–12^, which employed topology optimization inverse design with the Kreisselmeier–Steinhauser (K–S) aggregation function, we present a high-efficiency, broadband, achromatic imaging photonics element operating in the mid-infrared range (2–5 μm). The element comprises two achromatic metalenses, one focusing and the other reflective. In general, achromatic metalenses, whether focusing or reflective, have the capacity to focus light of varying wavelengths onto a single plane, thereby circumventing the blurring and color distortion that would otherwise occur and achieving full-color imaging. This is particularly the case with mid-infrared metalenses, which will have a significant impact on optical imaging, according to their characteristics, such as flatness, compactness and multispectral acquisition. Such developments will have a profound impact on imaging applications, including polarization-dependent imaging, wide-band focusing imaging and light-field imaging^13–18^. However, the meta-lens exhibits reduced efficiency under very high NA conditions^19^. Consequently, we demonstrate a high efficiency mid-infrared broadband (2–5 μm) achromatic focusing and reflective meta-lens under very high NA conditions (NA = 0.9), based on the previous inverse design methodology. The final design is derived from the initial metalens design through a reformulation of the optimization problem itself. This reformulation targets multi-focus points for the optimized structure, based on a double aggregation objective function. The optimization problem is first aggregated along the dimensions for different focus point positions, and then aggregated along the wavelength band. The design, based on the double aggregation method, is a longitudinal bifocal achromatic metalens. Despite the inverse design method overcoming the limitation of poor efficiency under very high NA conditions of the conventional design based on any phase modulation method (e.g. propagation phase, resonance phase, geometric phase), it still has a single focal point and is therefore limited in its applications^20,21^. The bifocal metalens has been designed to focus light waves at different longitudinal and transverse positions, thereby enhancing its versatility in optical systems. This functionality renders it highly suitable for advanced applications such as optical tomography, where precise light focusing is of paramount importance for imaging tissues^22^. Furthermore, the bifocal metalens is of significant value in virtual reality (VR) for the generation of an immersive visual experience^23,24^, and in micro imaging for the attainment of high-resolution images of microscopic structures. Previous research has demonstrated the realization of bifocal metalenses both theoretically^25^ and experimentally^26,27^. However, despite this advancement, the phase profile of such metalenses remains wavelength-dependent, exhibiting chromatic behavior. This limitation highlights the necessity for further optimization to achieve achromatic performance across a broader spectrum. A novel optimization problem and sensitivity adjoint were employed to design a high-efficiency achromatic broadband longitudinal bifocal metal lens in the mid-infrared (2–5 μm). The recent design and fabrication of metalenses on low-contrast dielectric materials, which allows for their transparent window to fall into the designed wavelength range, represents a significant advancement in the field of optics. This development has inspired numerous previously unimagined applications. Silicon nitride $\left( {Si_{3} N_{4} } \right)$ is a promising candidate for use as a fully CMOS-compatible platform for a wide range of applications across multiple wavelength ranges in both the visible and mid-infrared regions. Nevertheless, silicon dioxide (SiO₂) and silicon are employed in numerous photonics applications. The higher linear refractive index of $Si_{3} N_{4}$ enables robust optical confinement within the confines of a waveguide, while the broader band of Si₃N₄ than Si mitigates the likelihood of two-photon absorption and the associated free-carrier absorption. With its superiority of extremely low optical propagation losses, full CMOS compatibility, silicon nitride-based meta-lens is emerging as an exceptional solution in these respects^28^, so all the designs in this work are based on $Si_{3} N_{4}$ material from the range ($2\mu m :5\mu m$), where it’s provided transparence behavior along this range.

## Methods

### Working principle

**Fig. 1:**
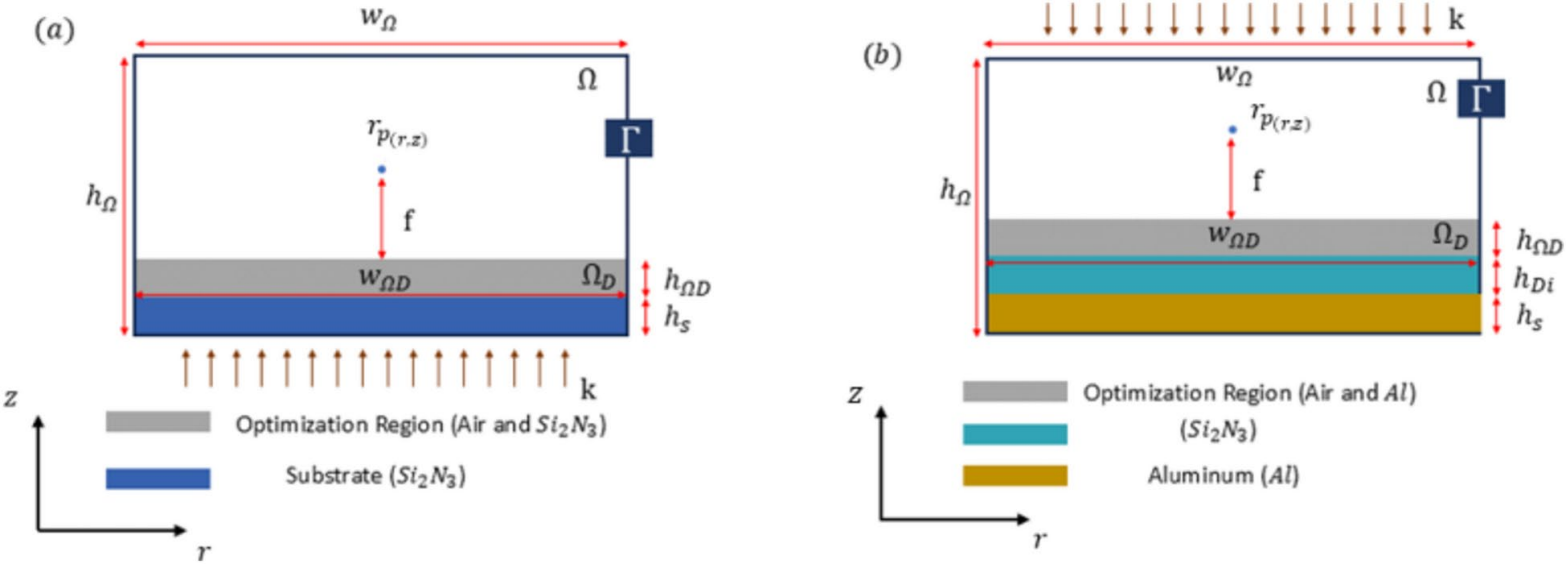
(**a**) The focusing meta-lens design and the boundary condition; (**b**) The Reflective meta-lens design and the boundary condition.

Figure 1 depicts the model physics within domain $\Omega$, with the boundary $\Gamma$. By employing Maxwell’s equation with the assumption of time-harmonic temporal behavior, we can identify a subset of $\Omega$ as the design domain, which we denote as ${\Omega }_{D}$. In light of this assumption, a two-dimensional Helmholtz-type partial differential equation is formulated for the out-of-plane component of the electric field within the domain $\Omega$.

1 $$\frac{1}{r}\frac{\partial }{\partial r} \left(r\frac{\partial E\left(r,z\right)}{\partial r}\right)+\frac{{\partial }^{2}E(r,z)}{\partial {z}^{2}}+{k}^{2}{\epsilon }_{r}\left(r,z\right) E\left(r,z\right)=F\left(r,z\right), \left(r,z\right) \in\Omega \subset {\mathbb{R}}^{2}$$

The electric field is represented by $$E$$. The wavenumber with wavelength $$\lambda$$ is given by $$k=2\pi /\lambda$$, where $$k$$ is the wavenumber and $$\lambda$$ is the wavelength. The forcing term $$F$$ is used to introduce an incident plane wave. The models depicted in Fig. 1a,b represent the focusing metalens and reflective metalens, respectively. Applying the first-order absorbing boundary conditions on all four exterior boundaries ($$n\nabla E\left(r,z\right)=-ikE\left(r,z\right), r\in\Gamma$$), where n denotes the surface normal and $$i$$ the imaginary unit. The focusing metalens is comprised of the model domain $$\Omega$$, which has a height of $${h}_{\Omega }$$. This domain is constructed from a substrate height of $${h}_{s}$$, an optimized region height of $${h}_{\Omega D}$$, and a remaining height for air. The width of the simulation is designated as $${w}_{\Omega }$$, while the width of the design is denoted as $${w}_{\Omega D}$$. The focus point, designated as $${r}_{p}$$, is subsequently determined, thereby enabling the field strength to be ascertained and the objective function, designated as $$\Phi$$, or figure of merit (FOM), to be calculated. This is achieved by generating an incident plane wave ($$k)$$, at the base of the model. The second design employs a metal-dielectric-metal (MDM) three-layer structure, wherein MDM meta-surfaces achieve high reflectivity due to the inherent reflective properties of metals such as gold, silver, and aluminum. These metals exhibit exceptional reflectivity across specific wavelength ranges, particularly in the visible and infrared spectra. Additionally, the metal layer functions as a back-reflector, ensuring that any light transmitted through the dielectric meta-elements is reflected through them. This double-pass mechanism significantly enhances reflection efficiency, as the light interacts with the meta-structured surface twice, thereby maximizing the overall reflectivity. The incident plane wave at the top of the model is denoted by $$k$$. The model employs the same absorbing boundary condition as the initial model, represented by $$\Gamma$$. Aluminum (Al) is utilized as the metallic component in the design, where the third layer possesses a height $${h}_{s}$$​ and a width equal to the model width $${w}_{\Omega }$$. The optimization region, situated between the air and the aluminum, has width and height dimensions $${w}_{\Omega d}$$​ and $${h}_{\Omega D}$$​, respectively. The middle dielectric layer, composed of $$S{i}_{3}{N}_{4}$$​, features a height $${h}_{Di}$$ and a width $${w}_{\Omega }$$​. All dimensions of the design in Fig. 1a,b are listed in Tables 1 and 2. The model equation, boundary conditions are discretized by finite element method (FEM)^29^.

**Table 1:** Values for Quantities in Fig. 1a for focusing meta-lens.

$h_{{\Omega }}$	${w}_{\Omega }$	$w_{{\Omega {\text{D}}}}$	$h_{{\Omega {\text{D}}}}$	${h}_{\text{s}}$	$f$	$NA$
40 µm	40 µm	40 µm	1.5 µm	1.5 µm	9 µm	0.9

**Table 2:** Values for Quantities in Fig. 1b for reflective meta-lens.

${h}_{\Omega }$	${w}_{\Omega }$	$w_{{\Omega {\text{D}}}}$	$h_{{\Omega {\text{D}}}}$	${h}_{\text{s}}$	${h}_{Di}$	$f$	$NA$
40 µm	65 µm	65 µm	1.5 µm	1.5 µm	1.5 µm	15 µm	0.9

In density-based topology optimization, material interpolation algorithms are employed to establish a correspondence between variations in the design field and alterations in local spatial material properties within the physical model. The interrelationship among the refractive index (η), extinction coefficient ($K$), and relative electric permittivity (${\varepsilon }_{r}$) is expressed as follows:

2 $${\varepsilon }_{r}=\left({\eta }^{2}-{K}^{2}\right)-2i\eta K .$$

**Fig. 2:**
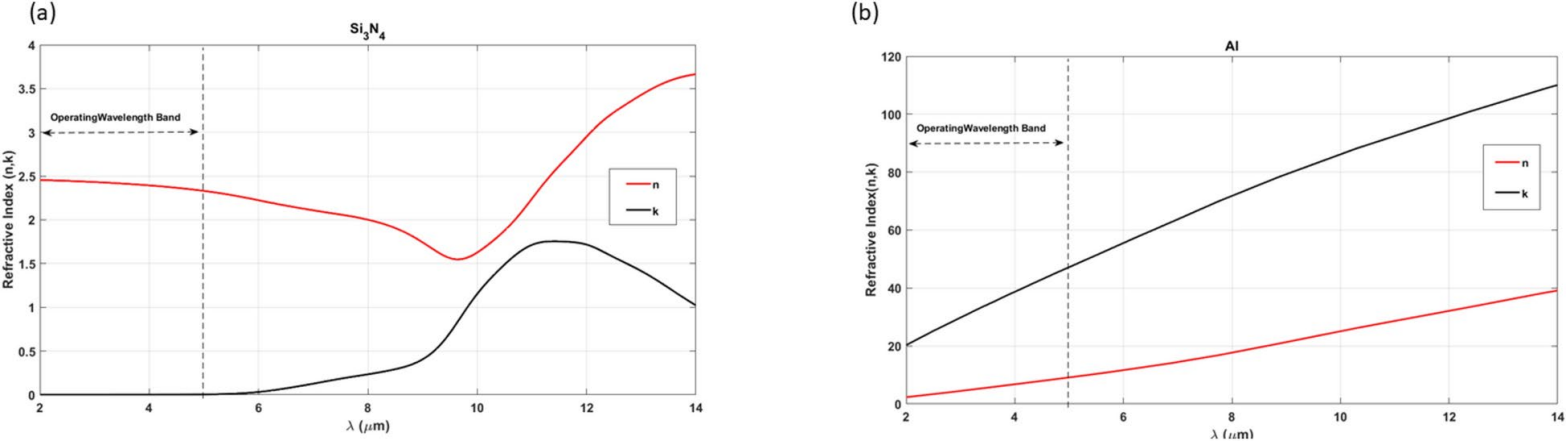
(**a**) The refractive index of $S{i}_{2}{N}_{3}$ along the range (2–5 µm); (**b**) The refractive index of $Al$ along the range (2 µm:5 µm).

To formulate the non-linear interpolation scheme^30^:

3 $$\begin{aligned} \epsilon_{r} \left( {\eta \left( \rho \right),K\left( \rho \right)} \right) = & \left( {\eta (\rho )^{2} - K(\rho )^{2} } \right) - {\text{i}}\left( {2\eta \left( \rho \right)K\left( \rho \right)} \right) \\ \eta \left( \rho \right) =\, & \eta_{{{\text{M}}_{1} }} + \rho \left( {\eta_{{{\text{M}}_{2} }} - \eta_{{{\text{M}}_{1} }} } \right) \\ K\left( \rho \right) =\, & K_{{{\text{M}}_{1} }} + \rho \left( {K_{{{\text{M}}_{2} }} - K_{{{\text{M}}_{1} }} } \right) . \\ \end{aligned}$$

where ${M}_{1}$ and ${M}_{2}$ denotes the two materials being interpolated, the interpolation parameters $\rho$ is varying from zero to one to relate between the two materials^31^. As illustrated in Fig. 2a, the refractive index of $S{i}_{3} {N}_{4}$ within the design bandwidth (2–5 μm) enables high transmittance, rendering it highly suitable for the focusing metal lens. In the case of the reflective meta-lens, aluminum is employed due to its exceptional reflectivity, as demonstrated in the metal-dielectric-metal structure shown in Fig. 2b. This is the primary rationale for conducting the entire optimization process within this bandwidth.

The figure of merit $\phi$ as a function of the magnitude ${\left|E\right|}^{2}$ at the focal point ${r}_{p}$ is:

4 $$\phi \left(\rho (r),{r}_{p}\right)={\left|E\left(\rho (r),{r}_{p}\right)\right|}^{2}={E\left(\rho (r),{r}_{p}\right)}^{*}E\left(\rho (r),{r}_{p}\right) .$$

where $\rho \left(r\right)$ describe a radially varying design field. The optimization problem based on The Kreisselmeier–Steinhauser (k–s)^32–34^ objective function targets 2 μm of wavelength range simultaneously with the number of points (${N}_{\lambda }=20$).

The standard formulation of Kreisselmeier–Steinhauser (k–s) aggregation function is defined as:

5 $$\phi \left(x\right)=\frac{1}{p}\text{ln}({\Sigma }_{i=1 }^{{N}_{\lambda } }{e}^{p {f}_{i}(x)})$$

Given that both the exponential and logarithmic functions are smooth and differentiable, the k–s aggregation function inherits these properties which is essential and particularly suit for gradient descent and any other gradient-based optimization methods.

The gradient of the k–s function with respect to $x$ can be derived using the chain rule. For an objective function ${f}_{i}(x)$, the gradient of the k–s function $\phi (x)$ is:

6 $$\nabla \phi \left(x\right)=\frac{{\Sigma }_{i=1 }^{{N}_{\lambda }}{e}^{p {f}_{i}(x)} \nabla {f}_{i}(x)}{{\Sigma }_{i=1}^{N}{e}^{p {f}_{i}(x)}}$$

This gradient is a weighted sum of the gradients of the individual objective functions, where the weights are determined by the relative magnitudes of ${e}^{p {f}_{i}(x)}$. This smooth blending of gradients contributes to stable convergence in gradient descent. The parameter $p$ in the k–s aggregation allows control over how closely function approximates the maximum of the objective functions. When $p$ is large, the k–s function behaves more like the maximum function, emphasizing the largest objective, when $p$ is small, the function behaves more like an average of the objectives. This controllability can help in fine-tuning the optimization process for better stability and convergence. Unlike the pure maximum or minimum function, which can be non-differentiable and lead to abrupt changes in the gradient, the k–s function provides a smooth transition between the objective functions. This smoothness prevents the optimizer from the encountering sudden changes in the gradient, which can destabilize the optimization process, but the most features we rely on for our designs is stability in response where the smooth nature of the k–s function ensures that small changes in $x$ lead to small changes in $\phi (x)$ and it is gradient, this predictability and smoothness contribute to a stable response during optimization. The optimizer is less likely to encounter sudden, unexpected changes in the objective landscape, leading to more reliable and stable convergence.

In some formulations, especially those dealing with constraints or when the objective is to be maximized, the k–s aggregation is written with a negative sign as:

7 $$\phi \left(x\right)=-\frac{1}{p}\text{ln}({\Sigma }_{i=1 }^{{N}_{\lambda } }{e}^{-p {f}_{i}(x)})$$

This formulation is suitable for our condition where the k–s function is used to aggregate constraint violations, the constraints are typically of the form ${g}_{i}\left(x\right)\underset{\_}{<}0$. TO combine these constraints into a single measure of constraint violation, the negative sign ensure that larger (more positive) constraint violations are penalized more heavily. In our problem we depend on maximization, where the higher values of ${f}_{i}\left(x\right)$ are better, using the negative exponential term ensures that the k–s function properly reflects the optimization direction. The term ${e}^{-p {f}_{i}(x)}$ reflects decreases for larger ${f}_{i}(x)$, thus when summed and processed through the logarithm, it correctly represents the objective in a form suitable for maximization. Achromatic behavior in a meta-lens means that the lens can focus light from different wavelengths to the same focal point with minimal chromatic aberration. The K–S aggregation function can help achieve this by balancing the light intensities across multiple wavelengths. By aggregating the intensities over different wavelengths, the optimization process aims to minimize the deviation in focal intensity for each wavelength where the K–S function penalizes large deviations in intensity for any wavelength, promoting a design where the focal point remains consistent across wavelengths. For multi-wavelength FOM (${\Phi }_{i}\left(E\left({\lambda }_{i},{r}_{P},{\varepsilon }_{r}\left(\rho \left(r\right),{\lambda }_{i}\right)\right)\right)$), the formulation of FOM based on the k–s objective function can be written as:

8 $${\Phi }_{k-S}=-\frac{1}{p}\text{ln}\left(\sum_{i=1}^{{N}_{\lambda }} {e}^{-p\left({\Phi }_{i}\left(E\left({\lambda }_{i},{r}_{P},{\varepsilon }_{r}\left(\rho \left(r\right),{\lambda }_{i}\right)\right)\right)\right.}\right) .$$

The equality constraints of the optimization problem are related to the operator ${{\ell}}_{EM },$ where the operator denotes applying the effect of the physical system to the state field for the excitation $(F$(${{\ell}}_{EM }\left(E\right)= \frac{1}{r}\frac{\partial }{\partial r} \left(r\frac{\partial E\left(r,z\right)}{\partial r}\right)+\frac{{\partial }^{2}E(r,z)}{\partial {z}^{2}}+{k}^{2}{\epsilon }_{r}\left(r,z\right) E\left(r,z\right)=F\left(r\right)$)). The solution to the optimization problem depends mainly on the interpolation parameters $\rho \left(r\right)$ to interpolate between the high index material ($S{i}_{3}{N}_{4}$) or ($Al$) and the low index material (air). To limit the spatial oscillation of the design field a standard filtering is applied to the $\rho$ parameter over ${\Omega }_{\text{D}}$ using the equation^35^:

9 $$- \left( {\frac{{r_{f} }}{2\sqrt 3 }} \right)^{2} \nabla^{2} \tilde{\rho }\left( {\mathbf{r}} \right) + \tilde{\rho }\left( {\mathbf{r}} \right) = \rho \left( {\mathbf{r}} \right),\;\;\;\;r_{f} > 0, r \in\Omega _{{\text{D}}} .$$

where the ${r}_{f}$ is the filter radius. Then the filter is followed by thresholding using a smoothed approximation of the Heaviside function (Eq. 10) to recover a design between the $S{i}_{3}{N}_{4}$ material and the background material (Air):

10 $${\bar{\tilde{\rho }}}=\frac{\text{tanh}(\beta \cdot \eta )+\text{tanh}(\beta \cdot (\widetilde{\rho }-\eta ))}{\text{tanh}(\beta \cdot \eta )+\text{tanh}(\beta \cdot (1-\eta ))}, \, \beta \in [1,\infty [, \eta \in \left[\text{0,1}\right].$$

where $\beta$ is the threshold strength and $\eta$ is the threshold level. The algorithm used to solve the design problem is MATLAB’s fmincon with all details in [supplementary information]. Finally, the optimization problem is formulated as:

11 $$\begin{aligned} & \mathop {max}\limits_{{\mathop {\tilde{\rho }}\limits^{ \_\_} }} \left( {\frac{ - 1}{p}{\text{ln}}\left( {\mathop \sum \limits_{{{\text{i}} = 1}}^{{\text{N}}} e^{{ - p\left( {{\Phi }_{{\text{i}}} \left( {E\left( {\lambda_{{\text{i}}} ,r_{p} ,\varepsilon_{r} \left( {{\bar{\tilde{\rho }}} \left( r \right),\lambda_{{\text{i}}} } \right)} \right)} \right.} \right.}} } \right)} \right) \\ & {\text{s}}{\text{.t}}{. }\left. {\ell_{EM} \left( {E\left( {\lambda_{{\text{i}}} ,r} \right),\varepsilon_{r} \left( {{\bar{\tilde{\rho }}} \left( r \right),\lambda_{{\text{i}}} } \right)} \right) = F\left( {r,\lambda_{{\text{i}}} } \right)} \right) \\ & \left. {\varepsilon_{r} \left( {{\bar{\tilde{\rho }}} \left( r \right),\lambda_{{\text{i}}} } \right) = \left( {\eta^{2} \left( {{\bar{\tilde{\rho }}} \left( r \right),\lambda_{{\text{i}}} } \right) - k^{2} \left( {{\bar{\tilde{\rho }}} \left( r \right),\lambda_{{\text{i}}} } \right)} \right) - 2{\text{i}}\eta \left( {{\bar{\tilde{\rho }}} \left( r \right),\lambda_{{\text{i}}} } \right)k\left( {{\bar{\tilde{\rho }}} \left( r \right),\lambda_{{\text{i}}} } \right)} \right) \\ & \eta \left( {{\bar{\tilde{\rho }}} \left( r \right),\lambda_{{\text{i}}} } \right) = \eta_{M1} \left( {\lambda_{{\text{i}}} } \right) + \rho \left( r \right)\left( {\eta_{M2} \left( {\lambda_{{\text{i}}} } \right) - \eta_{M1} \left( {\lambda_{{\text{i}}} } \right)} \right) \\ & k\left( {{\bar{\tilde{\rho }}} \left( r \right),\lambda_{{\text{i}}} } \right) = k_{M1} \left( {\lambda_{{\text{i}}} } \right) + \rho \left( r \right)\left( {k_{M2} \left( {\lambda_{{\text{i}}} } \right) - k_{M1} \left( {\lambda_{{\text{i}}} } \right)} \right) \\ & \mathop { \tilde{\rho }}\limits^{ \_\_} = \frac{{{\text{tanh}}\left( {\beta \cdot \eta } \right) + {\text{tanh}}\left( {\beta \cdot \left( {\tilde{\rho } - \eta } \right)} \right)}}{{{\text{tanh}}\left( {\beta \cdot \eta } \right) + {\text{tanh}}\left( {\beta \cdot \left( {1 - \eta } \right)} \right)}}, \\ & \quad \quad - \left( {\frac{{r_{f} }}{2\sqrt 3 }} \right)^{2} \nabla \tilde{\rho }\left( {\text{r}} \right) + \tilde{\rho }\left( {\text{r}} \right) = \rho \left( {\text{r}} \right), \\ & \quad \quad 0 \le \rho \left( r \right) \le 1,r \in\Omega _{{\text{D}}} . \\ \end{aligned}$$

The sensitivity of the k–s aggregation function (Eq. 9) respected to the design variable can be written as:

12 $$\frac{{\partial {\Phi }_{k - s} \left( {{\bar{\tilde{\rho }}} } \right)}}{{\partial {\bar{\tilde{\rho }}} }} = \frac{{\mathop \sum \nolimits_{i = 1}^{{N_{\lambda } }} e^{{ - p\left( {{\Phi }_{i} \left( {{\bar{\tilde{\rho }}} } \right)} \right)}} \frac{{\partial {\Phi }_{i} \left( {{\bar{\tilde{\rho }}} } \right)}}{{\partial {\bar{\tilde{\rho }}} }}}}{{\mathop \sum \nolimits_{i = 1}^{{N_{\lambda } }} e^{{ - p\left( {{\Phi }_{i} \left( {{\bar{\tilde{\rho }}} } \right)} \right)}} }}.$$

The gradient $\Phi$ respected to the design variables k $({{\bar{\tilde{\rho }}}}_{\text{k}})$ is derived by the adjoint sensitivity method^29^:

13 $$\frac{{\partial \Phi_{i} \left( {{\bar{\tilde{\rho }}} } \right)}}{{\partial {\bar{\tilde{\rho }}} }} = 2\Re \left[ {\lambda^{T} \frac{\partial S}{{\partial {\bar{\tilde{\rho }}} }}E_{i} } \right] .$$

where $S$, $\lambda$ are a vector related to nodal complex Lagrange multipliers and $\mathfrak{R}$ denotes the real part where the ${E}_{i}$ is the electric field for each wavelength.

The optimization parameters for the focusing and reflective meta-lenses are listed in the Tables 3 and 4, where the ${\rho }_{o}$ is the initial design field, where the initial design field, denoted as $\rho_{{\text{o}}}$, is initially chosen as 0.5, serving as an initial estimate between the high and low index interpolation values of 0 and 1. Throughout the iterative process, the design field is adjusted in accordance with the constraints imposed by the filter and thresholding conditions. This process continues until the design converges to an optimal solution, where the final design is constrained to only two possible values: 0 and 1.

**Table 3:** The optimization parameters for the focusing meta-lens.

Parameter	$\eta$	$\beta$	${r}_{f}$(nm)	${n}_{iter}$	$\text{p}$	${\rho }_{o}$
Value	$0.5$	$5$	60	50	5.37	0.5

**Table 4:** The optimization parameters for the reflective meta-lens.

Parameter	$\eta$	$\beta$	${r}_{f}$(nm)	${n}_{iter}$	$\text{p}$	${\rho }_{o}$
Value	$0.5$	$5$	60	50	4.73	0.5

Where the choice of $p$ values are observed in [supplementray information]. Where the ${n}_{iter}$ is the inner iteration taken to solve the optimization problem.

**Fig. 3:**
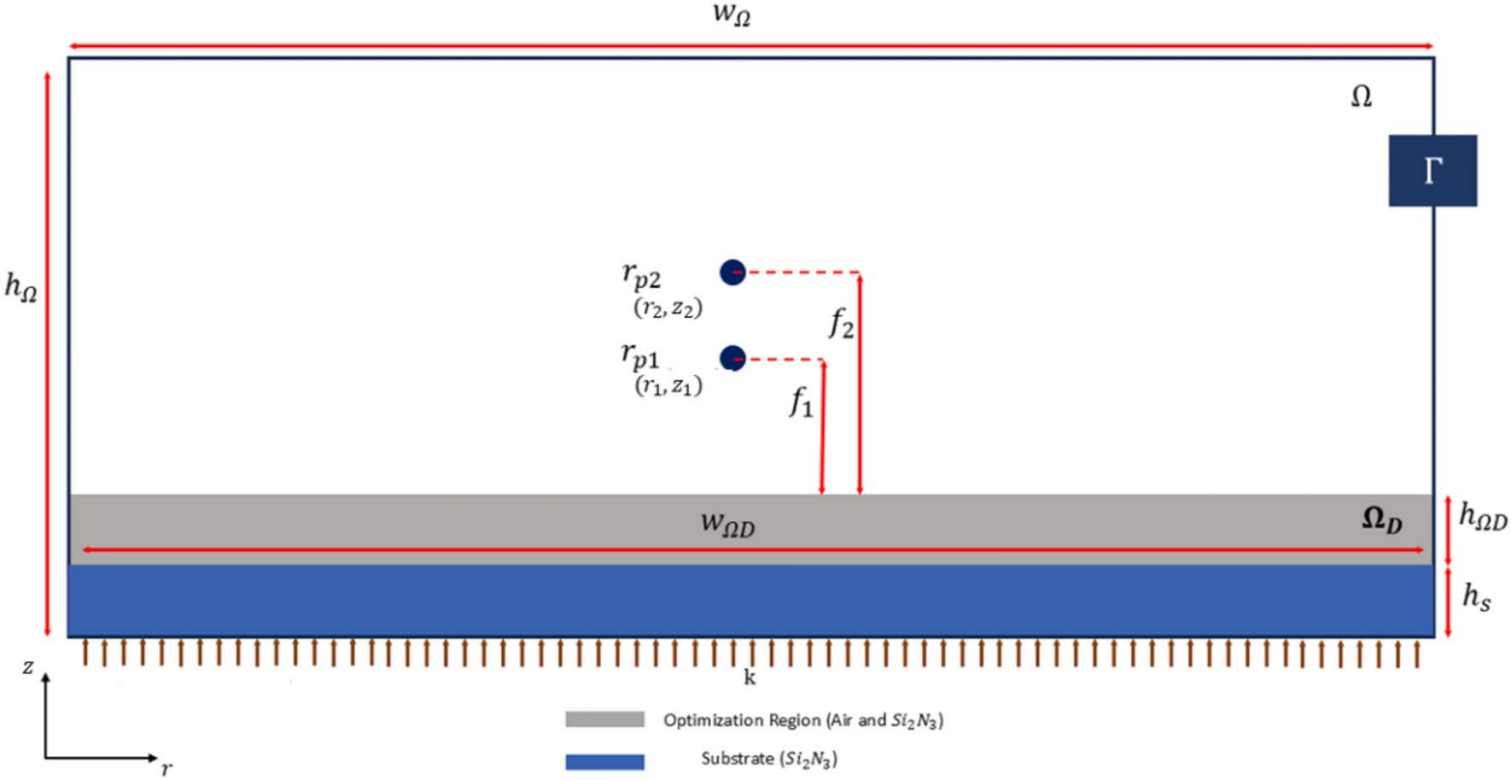
The multi-focal meta-lens design and the boundary condition.

The third design is longitudinal bifocal meta-lens where the bifocal capability of the meta-lens is achieved using a double k–s aggregation method. First aggregation (spatial focus) is applied in the spatial domain to ensure that the lens can focus light power at multiple focal points. This enables the lens to direct light into several distinct focal spots. With the same boundary condition and simulation parameters of the first design (focus meta-lens), the bifocal meta-lens is derived with two focus points vary along the z-direction (${r}_{p1} ,{r}_{p2}$) (Fig. 3). All the design parameters are listed in the Table 5

**Table 5:** Values for Quantities in Fig. 3 for multi-focal meta-lens.

${h}_{\Omega }$	${w}_{\Omega }$	$w_{{\Omega {\text{D}}}}$	$h_{{\Omega {\text{D}}}}$	${h}_{\text{s}}$	${r}_{{p}_{1}}$	$N{A}_{1}$	${r}_{{p}_{1}}$	$N{A}_{2}$
40 µm	50 µm	40 µm	1.5 µm	1.5 µm	25 µm, 11 µm	0.9	25 µm, 13 µm	0.88

In the context of designing a bifocal meta-lens, the k–s aggregation method helps to integrate multiple focusing objectives into a single scalar objective that can be optimized efficiently. The design specifies several target focal points (${N}_{p}$) where the light should be concentrated. These points are characterized by their positions in space relative to the meta-lens. For smaller value of $p$, the k–s function averages the intensities, which smooths out the variations and promotes a more uniform distribution. This balancing act ensures that no single focal point dominates the light distribution, and their intensities are collectively considered. To formulate the first aggregation k–s function for the multi-focus meta-lens, we need to define ${I}_{i , j}\left(E\left({\lambda }_{j},{{r}_{P}}_{i},{\varepsilon }_{r}\left(\rho \left(r\right),{\lambda }_{j}\right)\right)\right)$ where it represents the power intensity at the $i$-$th$ focal point for the $j$-$th$ wavelength, so we can write first aggregation as:

14 $$\phi_{j} = - \frac{1}{{p_{1} }} \ln \Sigma_{i = 1 }^{{N_{p} }} e^{{ - p_{1} I_{i , j} \left( {E\left( {\lambda_{j} ,r_{{P_{i} }} ,\varepsilon_{r} \left( {\rho \left( r \right),\lambda_{j} } \right)} \right)} \right)}}$$

Here, ${p}_{1}$ is a parameter controlling the sensitivity of the aggregation for the spatial focus points. The second aggregation integrates the results of the first aggregation across different wavelengths ($j=1 , 2, \dots ., {N}_{\lambda }$), so the overall aggregation is written as:

15 $$\phi _{{ks}} = - \frac{1}{{p_{2} }}\ln \Sigma _{{j = 1}}^{{N_{\lambda } }} e^{{ - p_{2} \left( {\phi _{j} } \right)}} = - \frac{1}{{p_{2} }}\ln \Sigma _{{j = 1}}^{{N_{\lambda } }} e^{{\left( {\frac{{p_{2} }}{{p_{1} }}\ln \Sigma _{{i = 1}}^{{N_{p} }} e^{{ - p_{1} I_{{i,j}} \left( {E\left( {\lambda _{j} ,r_{{P_{i} }} ,\varepsilon _{r} \left( {\rho \left( r \right),\lambda _{j} } \right)} \right)} \right)}} } \right)}}$$

Here, ${p}_{2}$ is a parameter controlling the sensitivity of the aggregation for the wavelengths. The double aggregation function consists of inner and outer aggregation. The inner aggregation (${\phi }_{j}$) relates to spatial focus points where each wavelength $j$, the k–s function aggregates at all spatial focal points $i$. This ensure that the design balances the field intensity across the spatial points for each individual wavelength which indirectly minimize the discrepancies between the intensities at different focal point (${I}_{1}= {I}_{2}=\dots {I}_{{N}_{p}}$). The outer aggregation relates to the wavelengths where the overall aggregation (${\phi }_{ks })$ then integrates this spatial aggregation across all wavelengths. This ensures that the design balances the light intensity distribution across different wavelengths, making the meta-lens achromatic broadband. To derive the sensitivity of the double aggregation k–s function respected to the design variables (${\bar{\tilde{\rho }}}$), we will follow these steps. Firstly, the gradient of the inner aggregation function to the design variables $(\frac{{\partial \phi }_{j}({\bar{\tilde{\rho }}})}{\partial {\bar{\tilde{\rho }}}} )$ is:

16 $$\frac{\partial {\phi }_{j}({\bar{\tilde{\rho }}})}{\partial {\bar{\tilde{\rho }}}} = \frac{{\Sigma }_{i=1 }^{{N}_{p}}{e}^{-{p}_{1} {I}_{i,j}({\bar{\tilde{\rho }}})}\frac{\partial {I}_{i,j}({\bar{\tilde{\rho }}})}{\partial {\bar{\tilde{\rho }}}} }{{\Sigma }_{i=1 }^{{N}_{p}}{e}^{-{p}_{1}{I}_{i,j}({\bar{\tilde{\rho }}})}},$$

Then, the gradient of the outer aggregation (overall aggregation) to the design variables ($\frac{{\partial \phi }_{k-s}({\bar{\tilde{\rho }}})}{\partial {\bar{\tilde{\rho }}}}$):

17 $$\frac{\partial {\phi }_{ks}({\bar{\tilde{\rho }}})}{\partial {\bar{\tilde{\rho }}}}=\frac{{\Sigma }_{j=1 }^{{N}_{\lambda }}{e}^{-{p}_{2}{\phi }_{j}({\bar{\tilde{\rho }}})}\frac{\partial {\phi }_{j }({\bar{\tilde{\rho }}})}{\partial {\bar{\tilde{\rho }}}}}{{\Sigma }_{j=1 }^{{N}_{\lambda }}{e}^{-{p}_{2}{\phi }_{j}({\bar{\tilde{\rho }}})}},$$

By substituting the $\frac{\partial {\phi }_{j}({\bar{\tilde{\rho }}})}{\partial {\bar{\tilde{\rho }}}}$ in the Eq. 17, we can write the sensitivity of the double aggregation k–s as:

18 $$\frac{\partial {\phi }_{ks}({\bar{\tilde{\rho }}})}{\partial {\bar{\tilde{\rho }}}}=\frac{{\Sigma }_{j=1 }^{{N}_{\lambda }}\left({e}^{-{p}_{2}{\phi }_{j}\left({\bar{\tilde{\rho }}}\right)}\frac{{\Sigma }_{i=1 }^{{N}_{p}}{e}^{-{p}_{1} {I}_{i,j}\left({\bar{\tilde{\rho }}}\right)}\frac{\partial {I}_{i,j}\left({\bar{\tilde{\rho }}}\right)}{\partial \stackrel{ }{{\bar{\tilde{\rho }}}}} }{{\Sigma }_{i=1 }^{{N}_{p}}{e}^{-{p}_{1}{I}_{i,j}\left({\bar{\tilde{\rho }}}\right)}}\right) }{{\Sigma }_{j=1 }^{{N}_{\lambda }}{e}^{-{p}_{2}{\phi }_{j}({\bar{\tilde{\rho }}})}}$$

The gradient $\Phi$ respected to the design variables ${\bar{\tilde{\rho }}}$ is derived by the adjoint sensitivity method:

19 $$\frac{{\partial I_{i,j} \left( {{\bar{\tilde{\rho }}} } \right)}}{{\partial {\bar{\tilde{\rho }}} }} = 2\Re \left[ {\lambda^{T} \frac{\partial S}{{\partial {\bar{\tilde{\rho }}} }}E_{i,j} } \right]$$

where $S$, $\lambda$ are a vector related to nodal complex Lagrange multipliers and $\mathfrak{R}$ denotes the real part and ${E}_{i,j}$ is electric field for each focus points ($i$) and wavelengths ($j$). The optimization problem primarily relies on the Sigmund code, with modifications to the solver for radial symmetry and adjustments to the objective function to align with our design requirements. Additionally, constraints on the slit widths have been tailored to suit the specifics of our case^29^. Same as the first optimization problem (Eq. 11), The equality constraints of the optimization problem are associated with the operator ${{\ell}}_{EM },$ which applies the physical system’s effect to the sate field for the excitation $F$, and the final optimization for the bifocal can be formulated as:

20 $$\begin{aligned} & \mathop {\max }\limits_{{\bar{\tilde{\rho }}}} \left( { - \frac{1}{{p_{2} }}~\ln \Sigma _{{j = ~~1}}^{{N_{\lambda } }} e^{{\left( {\frac{{p_{2} }}{{p_{1} }}~\ln \Sigma _{{i = 1~}}^{{N_{p} }} e^{{ - p_{1} ~I_{{i~,~~j}} \left( {E\left( {\lambda _{j} ,r_{{Pi}} ,\varepsilon _{r} \left( {\rho \left( r \right),\lambda _{j} } \right)} \right)} \right)}} } \right)}} } \right) \\ & s.t.\;~\left. {\ell _{{EM}} \left( {E\left( {\lambda _{i} ,r} \right),\varepsilon _{r} \left( {\bar{\tilde{\rho }}\left( r \right),\lambda _{i} } \right)} \right) = F\left( {r,\lambda _{i} } \right)} \right) \\ & \left. {\varepsilon _{r} \left( {\bar{\tilde{\rho }}\left( r \right),\lambda _{i} } \right) = \left( {\eta ^{2} \left( {\bar{\tilde{\rho }}\left( r \right),\lambda _{i} } \right) - k^{2} \left( {\bar{\tilde{\rho }}\left( r \right),\lambda _{i} } \right)} \right) - 2i\eta \left( {\bar{\tilde{\rho }}\left( r \right),\lambda _{i} } \right)k\left( {\bar{\tilde{\rho }}\left( r \right),\lambda _{i} } \right)} \right) \\ & \eta \left( {\bar{\tilde{\rho }}\left( r \right),\lambda _{i} } \right) = \eta _{{M1}} \left( {\lambda _{i} } \right) + \rho \left( r \right)\left( {\eta _{{M2}} \left( {\lambda _{i} } \right) - \eta _{{M1}} \left( {\lambda _{i} } \right)} \right) \\ & k\left( {\bar{\tilde{\rho }}\left( r \right),\lambda _{i} } \right) = k_{{M1}} \left( {\lambda _{i} } \right) + \rho \left( r \right)\left( {k_{{M2}} \left( {\lambda _{i} } \right) - k_{{M1}} \left( {\lambda _{i} } \right)} \right) \\ & \bar{\tilde{\rho }} = \frac{{\tanh \left( {\beta \cdot \eta } \right) + \tanh \left( {\beta \cdot \left( {\tilde{\rho } - \eta } \right)} \right)}}{{\tanh \left( {\beta \cdot \eta } \right) + \tanh \left( {\beta \cdot \left( {1 - \eta } \right)} \right)}}, \\ & \quad \quad - \left( {\frac{{r_{f} }}{{2\sqrt 3 }}} \right)^{2} \nabla \tilde{\rho }\left( r \right) + \tilde{\rho }\left( r \right) = \rho \left( r \right)~, \\ & \quad \quad 0 \le \rho \left( r \right) \le 1,r \in {\Omega }_{D} . \\ \end{aligned}$$

The optimization parameters for the multi-focus meta-lens Table 6 where the choice of ${p}_{1}$ and ${p}_{2}$ are provided in the [supplementary information].

**Table 6:** The optimization parameters for the bifocal meta-lens.

Parameter	$\eta$	$\beta$	${r}_{f}$(nm)	${n}_{iter}$	${p}_{1}$	${p}_{2}$	${\rho }_{o}$
Value	$0.5$	$5$	60	200	3.16	2.16	0.5

## Results

**Fig. 4:**
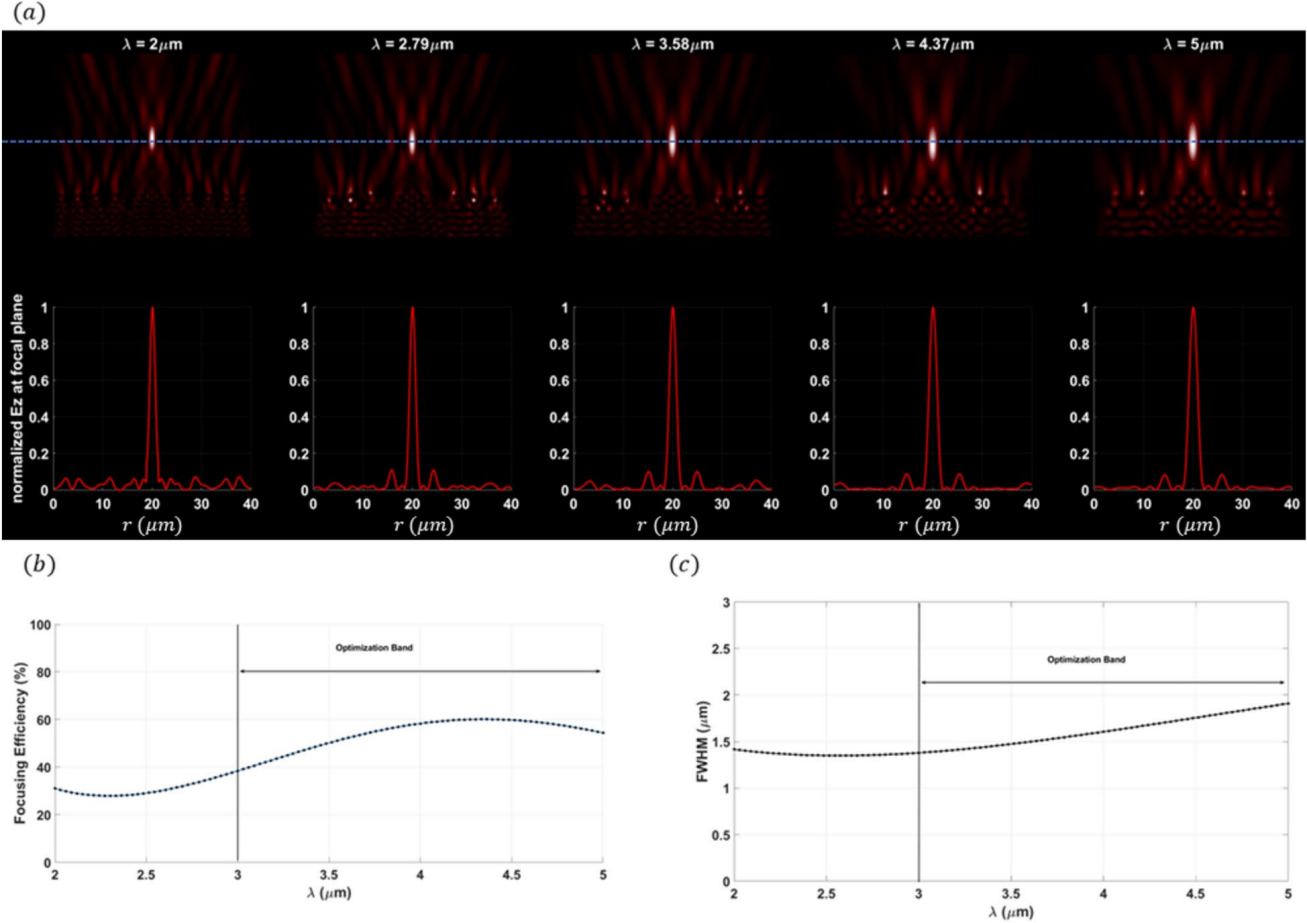
(**a**) The electric field distribution along the (r–z) at different wavelengths of the focusing meta-lens; (**b**) The focusing efficiency of the focusing meta-lens along the wavelength band (2 µm:5 µm); (**c**) The FWHM of the focusing meta-lens along the wavelength band (2 µm:5 µm).

**Fig. 5:**
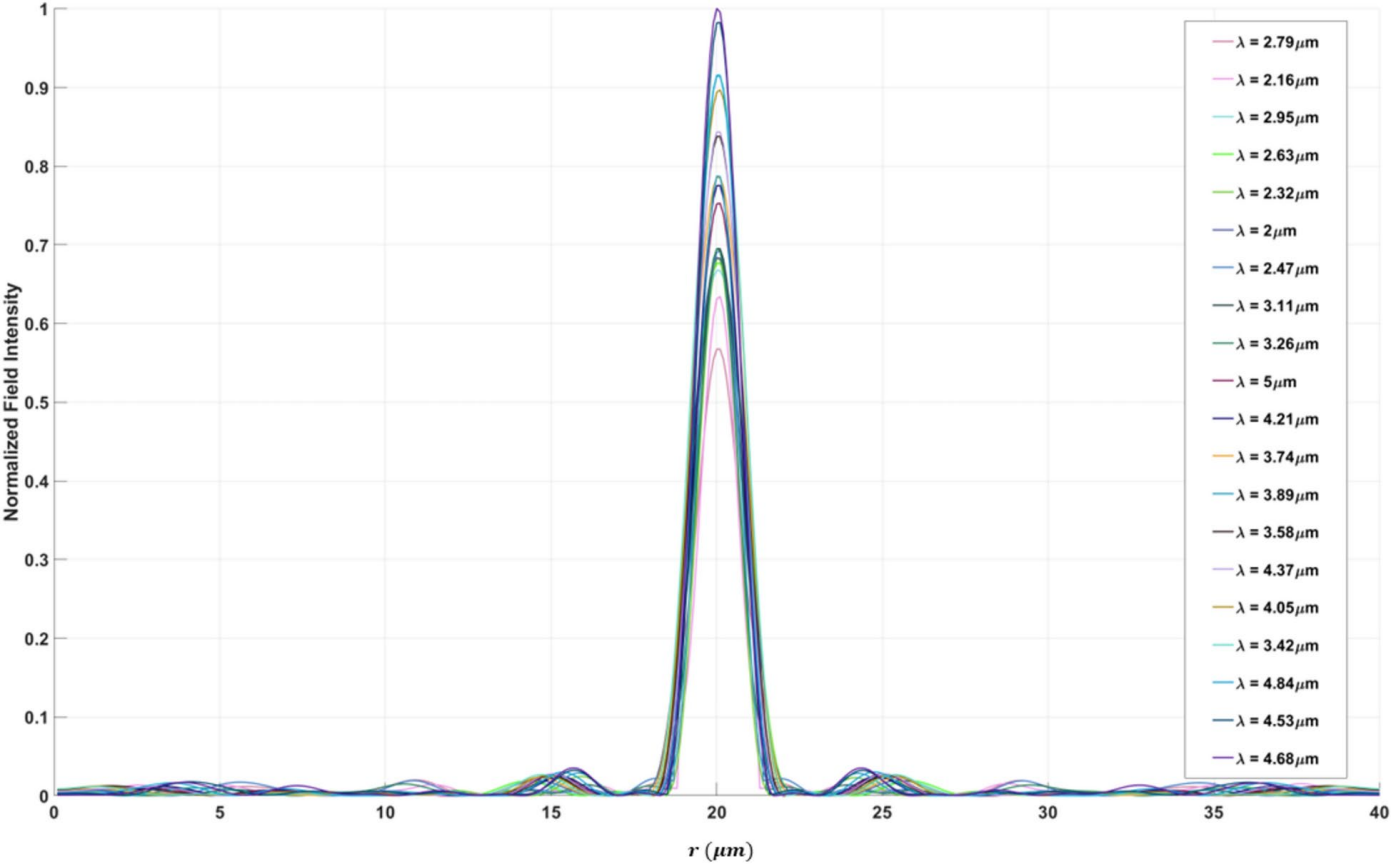
The normalized point spread function of the focusing meta-lens.

**Fig. 6:**
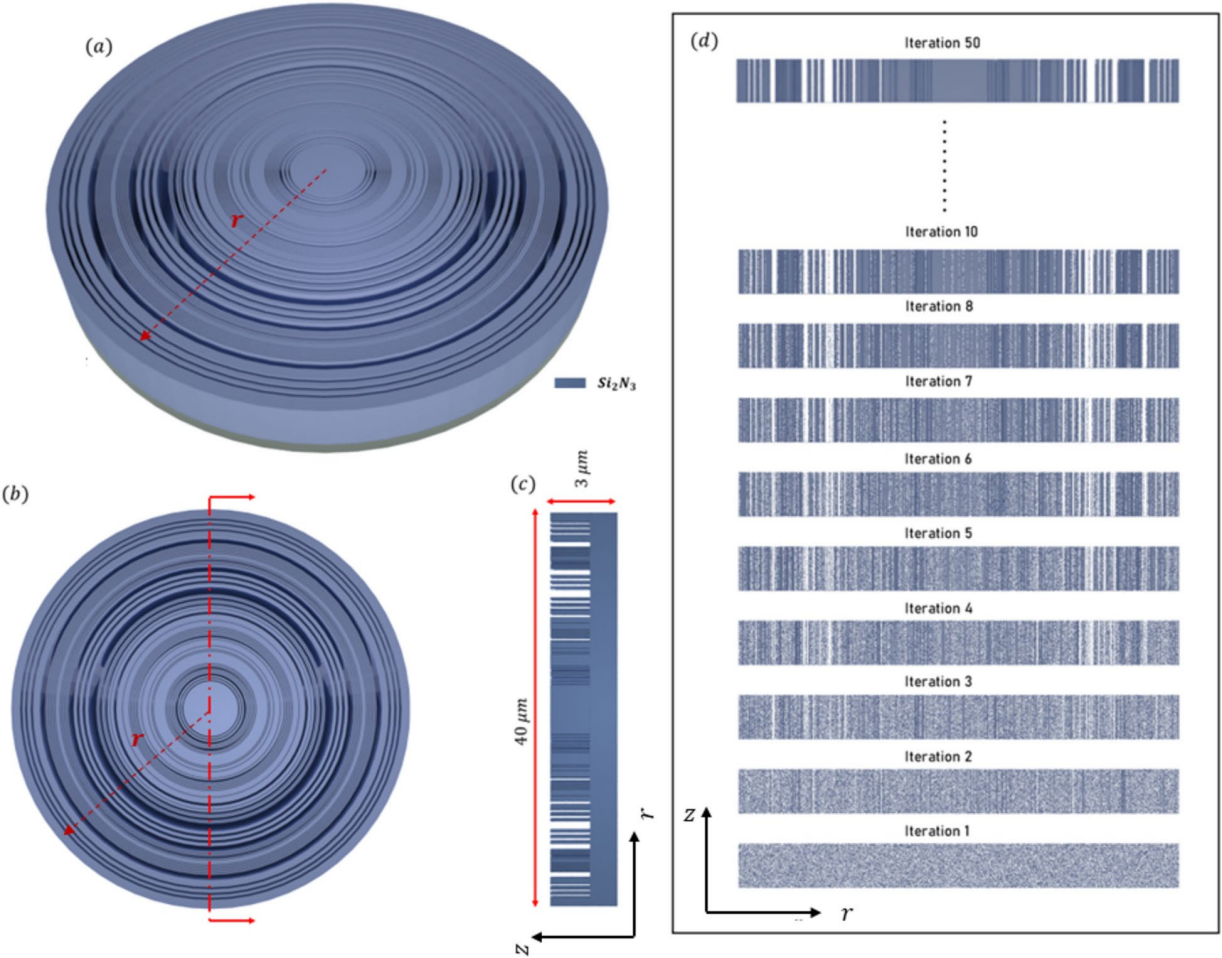
(**a**) 3D configuration of focusing the meta-lens assuming radial symmetry rendered and visualize by Vispy; (**b**) The top view of the 3D configuration of the focusing meta-lens; (**c**) Cross section of the structure in the r–z plane, where the blue represents the high index material ($S{i}_{2}{N}_{3}$) and the white represents the low index material (Air); (**d**) The geometry variation over the iteration starts from the first iteration after the initial condition directly (${\rho }_{o}$).

**Fig. 7:**
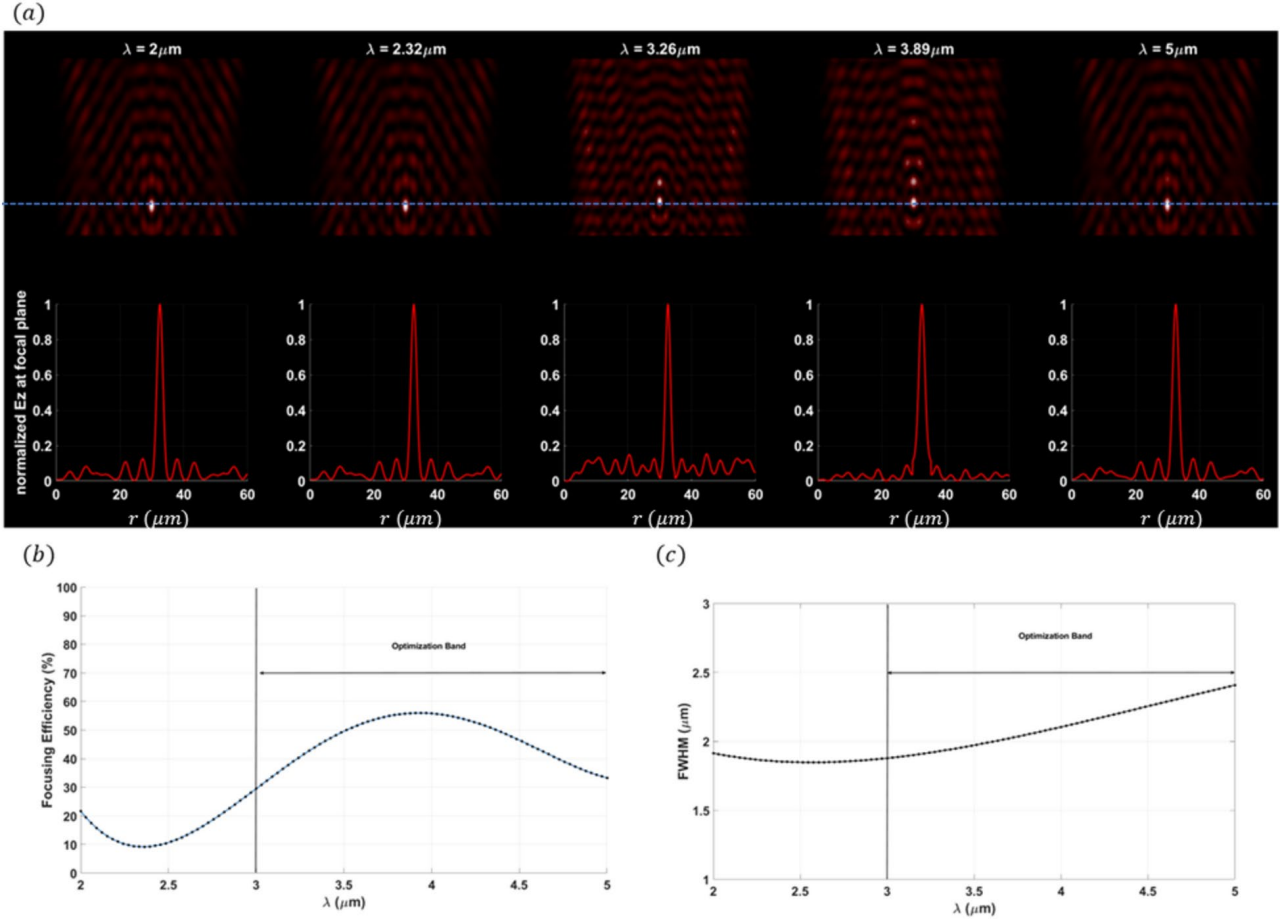
(**a**) The electric field distribution along the (r–z) at different wavelengths of the reflective meta-lens; (**b**) The focusing efficiency of the reflective meta-lens along the wavelength band (2 µm:5 µm); (**c**) The FWHM of the reflective meta-lens along the wavelength band (2 µm:5 µm).

**Fig. 8:**
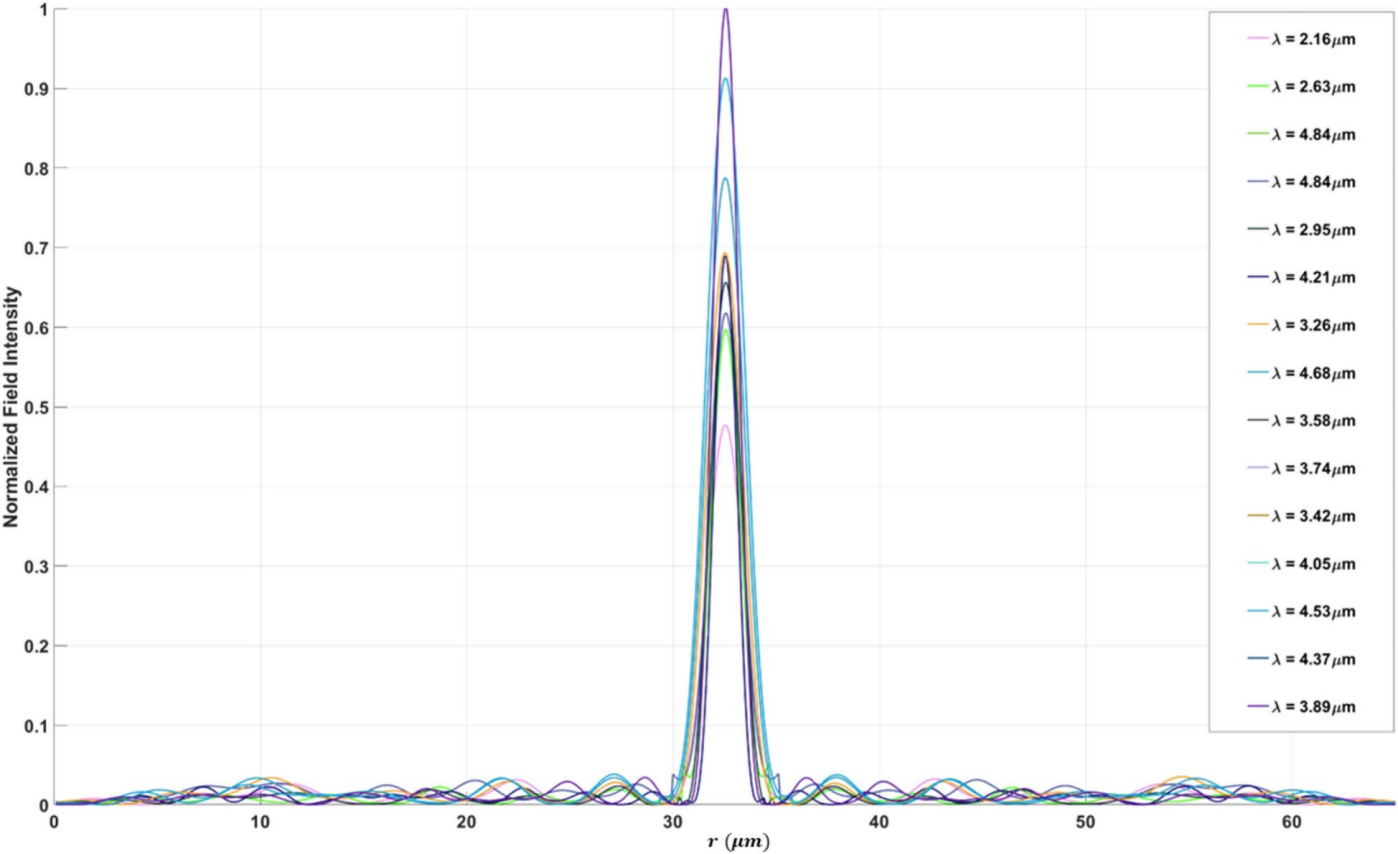
The normalized point spread function of the Reflective meta-lens.

**Fig. 9:**
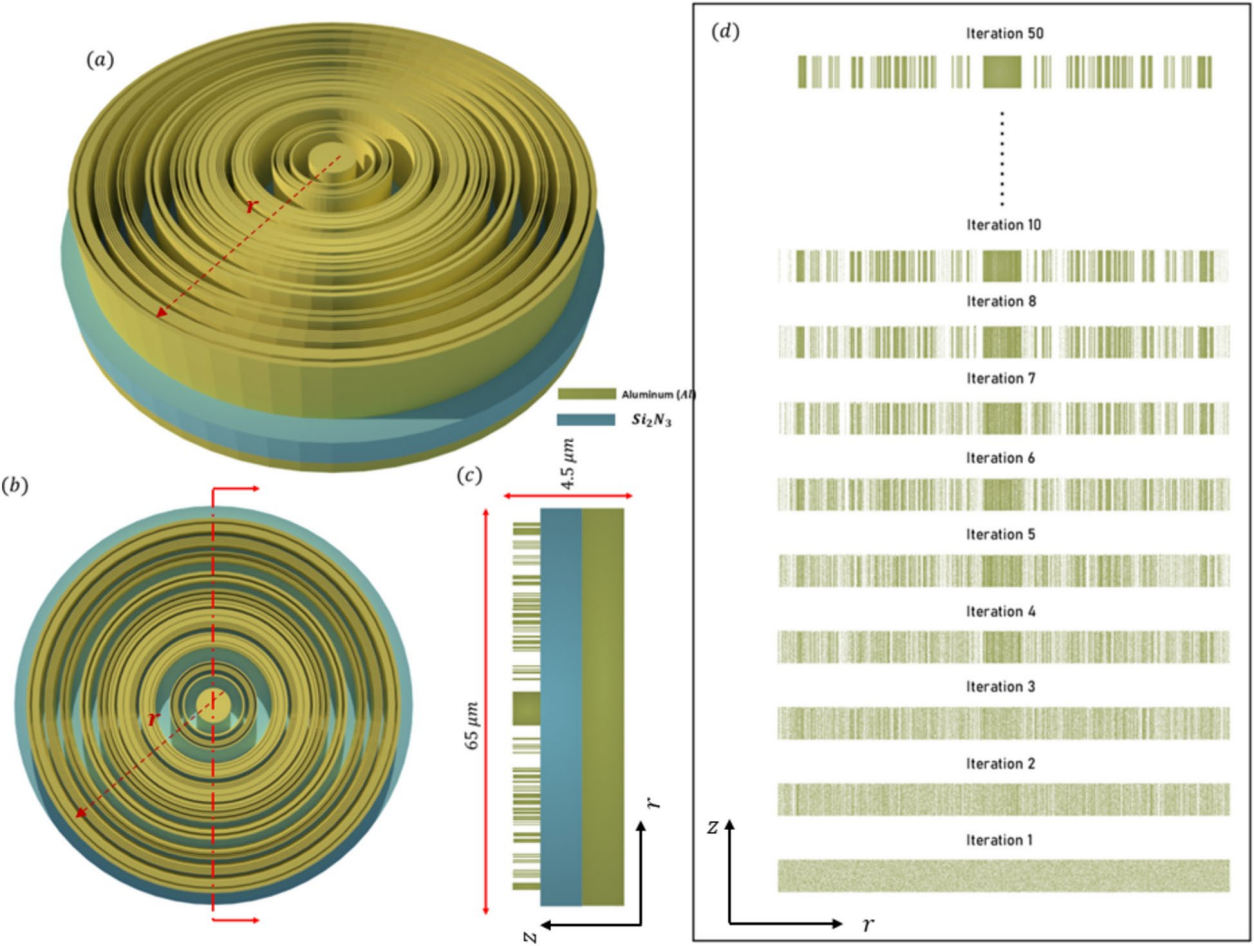
(**a**) 3D configuration of reflective meta-lens assuming radial symmetry rendered and visualize by Vispy; (**b**) The top view of the 3D configuration of the reflective meta-lens; (**c**) Cross section of the structure in the r–z plane, where the blue represents the $S{i}_{2}{N}_{3}$, yellow represents $Al$ and the white represents Air; (**d**) The geometry variation over the iteration starts from the first iteration after the initial condition directly (${\rho }_{o}$).

The efficiency and performance of a meta-lens are critically evaluated using several key parameters: focusing efficiency, full width half maximum (FWHM), field distribution along the meta-lens, and the point spread function (PSF). Focusing efficiency is a pivotal metric that quantifies the meta-lens’s ability to concentrate incident light into a focal point. This parameter is crucial for applications requiring precise light manipulation, such as high-resolution imaging and advanced optical sensing. The full width half maximum (FWHM) indicates the spread of the focused light spot; a smaller FWHM signifies a sharper focus, which is indispensable for achieving superior resolution in imaging applications. A well-defined PSF with minimal side lobes denotes a clean and concentrated focal spot, which is indicative of the meta-lens’s high resolution and efficiency. Maximizing the focusing efficiency through optimization process ensures that a greater proportion of light is confined to a minimal spatial region, thereby significantly enhancing the PSF’s intensity at the focal point. This optimized efficiency not only sharpens the PSF, resulting in a more pronounced and defined central peak but also minimizes the dispersion of light into side lobes, thereby substantially improving image resolution. Furthermore, by attenuating the intensity of these side lobes, which are regions where light is undesirably diffused, the meta-lens achieves superior image clarity and accuracy, effectively mitigating aberrations and reducing image artifacts. As the focusing efficiency increases, the PSF becomes sharper, which in turn decreases the FWHM. A smaller FWHM implies that the lens can resolve finer details, which is particularly important for applications requiring high precision, such as microscopy or imaging. During the optimization process, as the algorithm adjusts the design variables to maximize focusing efficiency, it inherently reduces the FWHM of the PSF. This is because the optimization is pushing the lens to concentrate light more effectively at the focal point, leading to a more confined and sharper PSF. Moreover, the field distribution along the meta-lens surface provides essential insights into the interaction of the light with the meta-surface. A uniform field distribution is paramount for ensuring consistent focusing performance across the entire lens. The point spread function further characterize the meta-lens’s response to a light source, serving as a critical indicator of its imaging performance. Additionally, the final structure of the meta-lens in 2d in is converted to 3D where The meta-lens structure in 3D can be generated by revolving the 2D axisymmetric design (defined in the *r*–*z* plane) around the *z*-*axis*. This creates a 3D structure without requiring explicit modeling in all three dimensions. In this study, we will use these parameters as the primary metrics for evaluating all the meta-lens designs presented in this paper, including focusing meta-lens, reflective meta-lens and multi-focal lens. By consistently applying these evaluation criteria, we aim to provide a comprehensive and comparative analysis of different meta-lens designs. We applied inverse design optimization within the wavelength range of $3 \mu m to 5 \mu m$ to refine the meta-lens structure. To extend the analysis to the 2–5 µm range, we employed a direct solver using the finite element method (FEM). In this approach, the final binarized structure was analyzed using strict binary values of 0 and 1. Although the filtering stages in the inverse design optimization process reduced most intermediate grayscale points to values near 0 or 1, the direct solver ensured that these values were precisely set to either 0 or 1, thereby guaranteeing the accuracy and fidelity of the final binary structure. The final binarized dataset is rendered and visualized using the Vispy 3D data visualization library^36^, ensuring high-performance graphical representation and precise spatial interpretation of the data. The performance of the focusing meta-lens is evaluated across these parameters, each elucidated based on empirical data and corresponding figures. Figure 4a presents the electric filed distribution along the (r-z) plane, illustrating variations in field intensity across the meta-lens surface and blue dashed line explains the focal plane where it’s indicated the stable convergence of the focus point along the wavelength design band (achromatic behavior). Figure 4b details the focusing efficiency across the wavelength band spanning from 2 to 5 µm. The average of the focusing efficiency is 46.32%, with a peak efficiency of 60.32%, demonstrating the meta-lens’s capability to efficiently concentrate light under very high NA = 0.9. The full width half maximum under the same wavelength range is depicted in Fig. 4c. Here, the average FWHM measures 1.53 µm, with a maximum of 1.91 µm, indicating the spatial extent of the focused light spot and its variability across the wavelengths. Figure 5 displays the normalized point spread function (PSF) of the meta-lens, depicting the intensity of the focused spot and its highlights the meta-lens’s imaging performance, showing a clean and concentrated focal point characteristic of high-resolution optics. The final optimized structure of the focusing meta-lens is presented is presented in Fig. 6, exhibiting radial symmetry in its 3d representation (Fig. 6a, top view Fig. 6b, cross-sectional view Fig. 6c), and the geometry variation over the optimization iteration process (Fig. 6d). These figures illustrated the engineered design aimed at maximizing optical performance through precise material distribution and geometric arrangement. The parameters for the reflective meta-lens include the field distribution along the (r-z) plane(Fig. 7a) and the achromatic behavior of the reflective meta-lens, focusing efficiency across the wavelength range from 2 to 5 µm (Fig. 7b), with an average of 36.1%, achieving a maximum efficiency of 55.98%. Figure 7c presents the FWHM variation, with an average of 2.04 µm and a maximum of 2.41 µm, highlighting the spatial extent of the focused light spot. Figure 8 displays the normalized point spread function (PSF), showing the intensity distribution of the focused spot, reflecting the meta-lens’s imaging performance. Figure 9 depicts the final optimized structure of the reflective meta-lens, comprising three distinct layers, the first layer facilities optimization and interpolation between the metal ($Al$) and air interfaces, the second layer consists of the dielectric material ($S{i}_{3}{N}_{4}$), crucial for manipulating light at the nano-scale. The third layer serves as the substrate, supporting the meta-else structure composed of $Al$. These layers are visualized in three perspectives. Figure 9a presents a full 3D view assuming radial symmetry, providing a comprehensive look at the entire meta-lens structure. Figure 9b offers a top view, emphasizing the layout and distribution of the different layers across the meta-lens. Figure 9c displays a cross-sectional view, revealing the detailed profile and layer composition along a specific axis and Fig. 9d shows the iterations of the optimized region of the reflective meta-lens. The designs of both the focusing and reflective meta-lens in this study are based on the advanced methodology of inverse design with topology optimization employing a k–s aggregation function. This approach represents a significant leap forward from conventional meta-lens design methods, offering efficiency and overcoming inherent limitations. Specifically, under high NA conditions in the mid-infrared wavelength range, the meta-lens designs benefit from enhanced focusing efficiency and resolution, crucial for applications requiring precise light control and detection. Recent advancements in meta-lens technology have highlighted the transformative impact of inverse design and topology optimization methodologies compared to conventional approaches. Conventional meta-lens designs often rely on heuristic or manual approaches, which may struggle to achieve optimal performance across diverse operational conditions, especially in mid infra-red^13,37,38^. In contrast, inverse design methodologies revolutionize meta-lens engineering by formulating the design process as an optimization challenge^19,39^. This approach enables precise tailoring of meta-lens structures to achieve specific optical functionalities, such as high-resolution imaging and efficient light manipulation. Our design ensures that the design not only meets but exceeds performance expectations, particularly under high numerical aperture (NA) conditions and in the mid-infrared wavelength range. These advancements in meta-lens design enable enhanced focusing efficiency, resolution, and overall optical performance, crucial for applications demanding precise light control and detection.

**Fig. 10:**
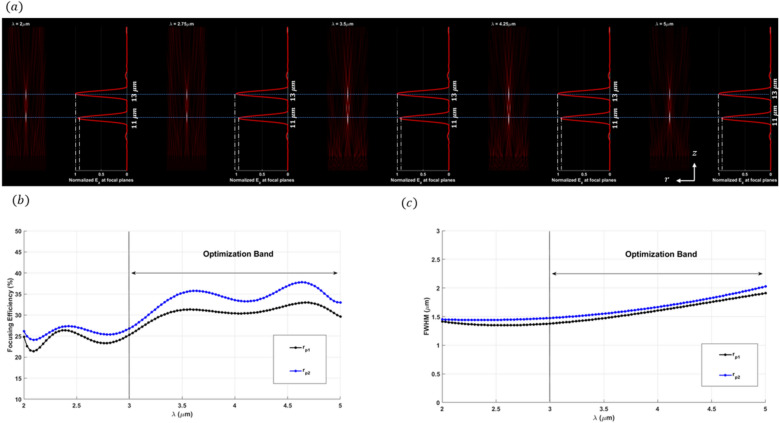
(**a**) The electric field distribution along the (r–z) at different wavelengths of the bifocal meta-lens; (**b**) The focusing efficiency of the bifocal meta-lens along the wavelength band (2 µm:5 µm); (c) The FWHM of the bifocal meta-lens along the wavelength band (2 µm:5 µm).

The final design (bifocal focal meta-lens), Fig. 10a Illustrates the field across the $(r-z)$ plane of the meta-lens. This distribution not only shows the spatial variation in field intensity but also highlights how the field intensity is distributed among two distinct focal points ($E$ intensity along the focal plane (blue dashed line)) showing no deviation from the focal plane as light converges at the focal points. This lack of deviation is a strong indication of the achromatic behavior of the meta-lens, meaning it can focus light of different wavelengths to the same focal point without dispersion. Such achromatic performance is highly beneficial for applications requiring precise and wavelength-independent focusing, including high-resolution imaging, multi-wavelength sensing, and broadband optical systems.

**Fig. 11:**
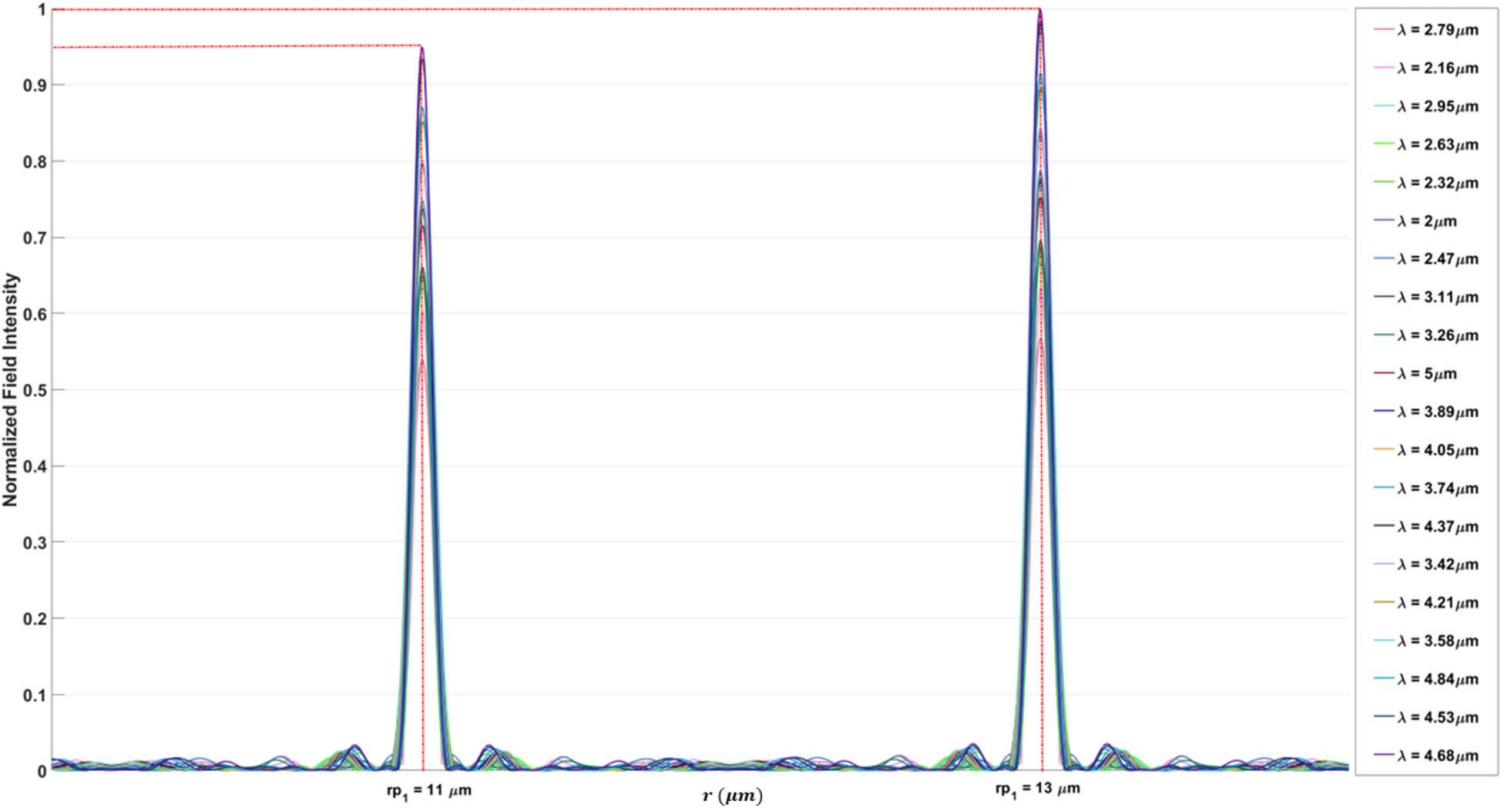
The normalized point spread function of the bifocal meta-lens.

**Fig. 12:**
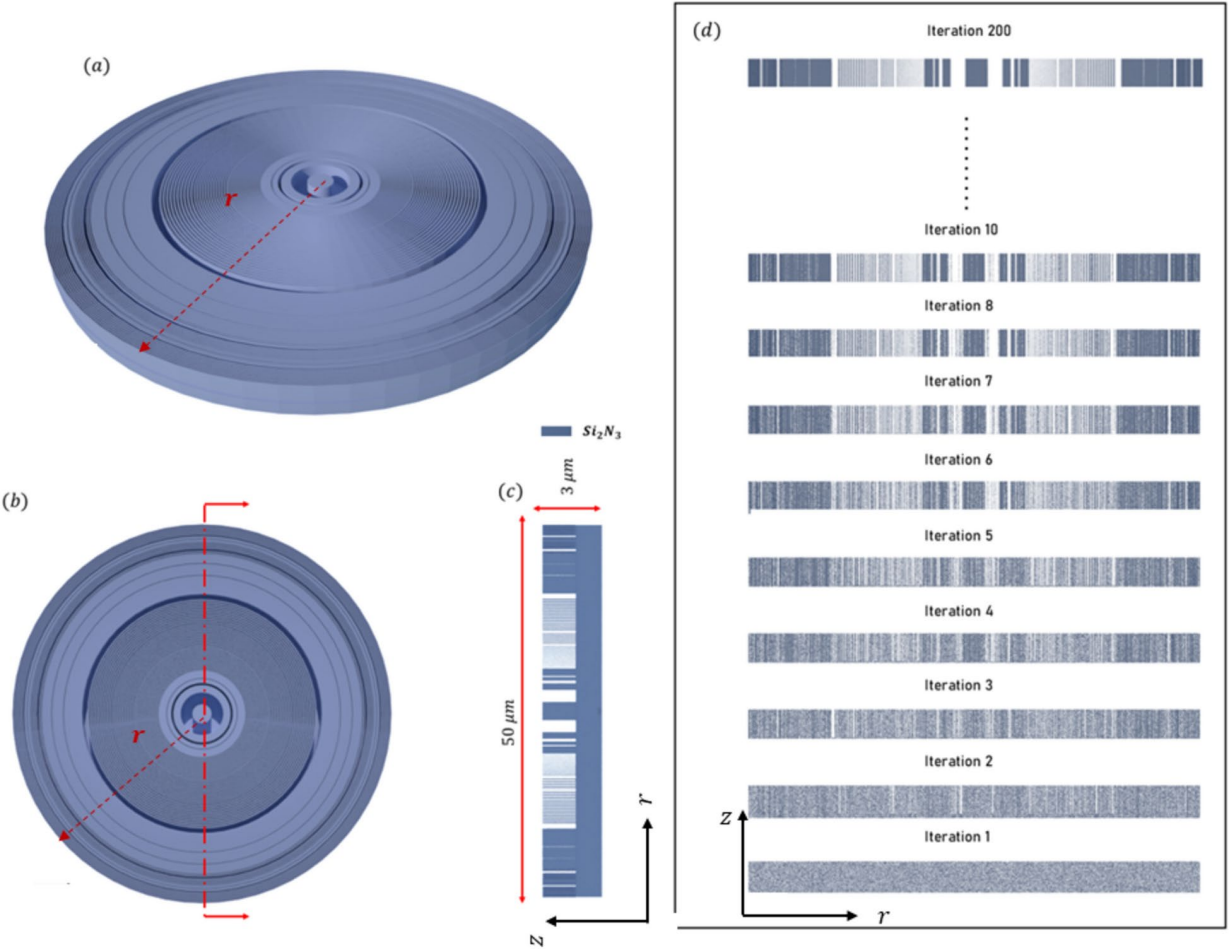
(**a**) 3D configuration of bifocal meta-lens assuming radial symmetry rendered and visualize by Vispy; (**b**) The top view of the 3D configuration of the bifocal meta-lens; (**c**) Cross section of the structure in the r-z plane, where the blue represents the $S{i}_{2}{N}_{3}$, and the white represents Air); (**d**) The geometry variation over the iteration starts from the first iteration after the initial condition directly (${\rho }_{o}$).

Figure 10b displays the focusing efficiency of the bifocal meta-lens at two distinct focal points each characterized by different numerical aperture (NA), at the first focal point with an NA of 0.9, the maximum focusing efficiency was found to be 32.96%, with an average of 28.54%, In contrast, at the second focal point with an NA of 0.88, the maximum focusing efficiency increased to 37.97%, and the average focusing efficiency was recorded at 31.43%. The observed slight drop in efficiency between the two focal points can be attributed to the NA conditions. As the NA increases, the focusing efficiency decreases. This is due to the fact the higher NA values correspond to a larger acceptance angle for the meta-lens, which makes it more challenging to focus light efficiently due to increased aberrations and diffraction effects. Therefore, the bi-focal meta-lens exhibits slightly lower focusing efficiency at ${r}_{p1}$ compared to ${r}_{p2}$. The FWHM of the bifocal meta-lens (Fig. 10c) reveals significant differences between focal points characterized by different numerical apertures (NA). At the focal point with NA = 0.9, the FWHM exhibited a maximum value of $1.9 \mu m$ and an average value of 1.53 µm, while at NA = 0.88, the FWHM increased to a maximum of 2.02 µm, with an average value of 1.61 µm. The normalized point spread function (PSF) of the bifocal meta-lens is depicted in Fig. 11, illustrating its spatial distribution and focal characteristics, the normalized ratio between the two focal points, ${r}_{p1} :{r}_{p2}$ is 0.8:1, indicating a balanced intensity distribution across the focal plane, the presence of minimal side lobes in the PSF signifies a clean and concentrated focal spot, crucial for precise imaging and sensing applications. Figure 12 illustrates the multi-focal meta-lens design, with Fig. 12a showing the 3D configuration assuming radial symmetry, Fig. 12b presenting the top view, and Fig. 12c depicting the cross-section in the r-z plane where blue represents $S{i}_{3}{N}_{4}$ and white represents air. The optimized region of the bifocal-lens over the iteration is observed in Fig. 12d. The innovative methodology of using double aggregation in the design of bifocal meta-lenses presents a significant advancement over conventional and traditional methods^40^. This approach addresses the critical limitation of poor efficiency under very high NA conditions, which has been a president challenge in the field of photonics. The inverse design with topology optimization methodology integrates with k–s aggregation function with an additional layer of aggregation, allowing for the optimization of multiple focal points simultaneously while maintaining efficiency. Traditional multi-focal meta-lens designs often struggle with efficiency, particularly at high NAs, due to the inherent difficulty in managing the conflicting requirements of multiple focal points. The double aggregation methodology circumvents these issues by providing a more comprehensive optimization landscape. The dual aggregation approach ensures that the optimization process accounts for the interactions between multiple focal points and the high NA, leading to a design that maximizes efficiency across all focal points. This technique diverges from traditional meta-lens approaches, which often rely on heuristic or iterative optimization methods that fail to adequately handle the complex trade-offs between multiple focal points and high NA requirements. The results demonstrate a substantial improvement in focusing efficiency, with the meta-lens achieving near-ideal performance metrics even under extreme NA conditions. This represents a significant leap forward in the design of bifocal meta-lenses, providing a solution that is both effective and scalable. The implications of this methodology are profound for various applications in the photonics field high-efficiency bifocal meta-lenses are crucial for advanced imaging systems, optical communications techniques, and biomedical devices. The ability to design meta-lenses that maintain high efficiency at high NAs open up new possibilities for miniaturized optical systems and enhances the performance of existing technologies.

In imaging systems, for instance, the improved focusing efficiency and precision offered by the inverse design with topology optimization meta-lens can lead to higher resolution and better image quality, in optical communication, these meta-lenses can enhance signal clarity and reduce losses^41^, leading to more reliable and faster data transmission. For biomedical applications^42^, high efficiency meta-lens can improve the accuracy and effectiveness of diagnostic devices.

**Fig. 13:**
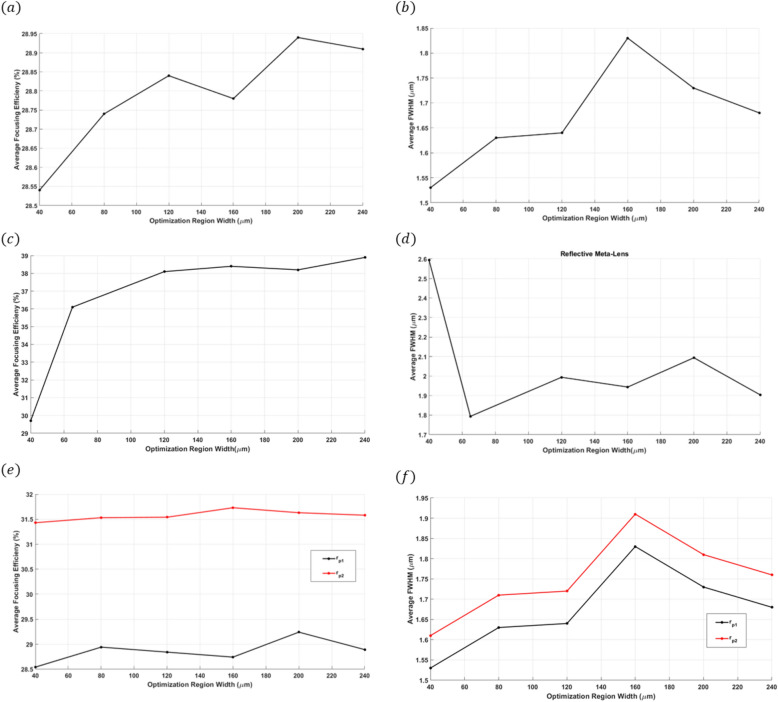
(**a**) The average focusing efficiency of the focusing meta-lens along the width from $10 \lambda$ to $60 \lambda$; (**b**) The average FWHM of the focusing meta-lens along the width from $10 \lambda$ to $60 \lambda$; (**c**) The average focusing efficiency of the reflective meta-lens along the width from $10 \lambda$ to $60 \lambda$; (**d**) The average FWHM of the reflective meta-lens along the width from $10 \lambda$ to $60 \lambda$; (**e**) The average focusing efficiency of the bi-focal meta-lens along the width $10 \lambda$ to $60 \lambda ;$ (**f**) The average FWHM of the bi-focal meta-lens along the width from $10 \lambda$ to $60 \lambda .$

To tackle the challenge of designing a meta-lens for large areas^43^, we performed optimization across various widths ranging from 10λ to 60λ. We then compared the average focusing efficiency and full width at half maximum (FWHM) across these different widths. For all three designs, the optimization region’s height ${h}_{\Omega }$ was set at 1.5 µm. As depicted in Fig. 13a,b, the average focusing efficiency and FWHM of the focusing meta-lens remain approximately consistent. Similarly, for the reflective meta-lens, the average focusing efficiency (Fig. 13c) and FWHM (Fig. 13d), which range from 65 to 240 µm, also show close agreement. Lastly, the bi-focal meta-lens exhibits comparable average values for focusing efficiency and FWHM, as shown in Fig. 13e,f.

A meta-lens designed for achromatic focusing might have a pattern of nanostructures that vary in shape across the lens surface. The two-photon polymerization fabrication method would be used to precisely fabricate these varying nanostructures^44^, creating a lens that focuses light across a broad spectrum of wavelengths to a single focal point. The axisymmetric nature of the lens would ensure uniform focusing performance regardless of the incident light angle.

## Conclusion

This paper presents a high efficiency broadband achromatic focusing and reflective meta-lens based on inverse design with topology optimization. The design depends mainly on the $S3{N}_{N}$ material in the wavelength range (2 µm:5 µm). The results revealed that the focusing efficiency is very high for all numerical aperture condition (NA = 0.9) with average efficiency 46.3% and 36.1% respectively. The bifocal presented in this study demonstrated remarkable capabilities at two distinct focal points characterized by NA of 0.9 and 0.88. This meta-lens achieves high efficiency under these high NA conditions, evidence by maximum focusing efficiencies of 32.96% and 37.96%, respectively, and average efficiencies exceeding 28% and 31% respectively. The methodology of inverse design with topology optimization, utilizing double k–s aggregation, represents a novel approach that has significantly enhanced the performance of the meta-lens, this methodology has overcome traditional design limitations by optimizing the structure to maximize efficiency and minimize aberrations under challenging optical conditions. Moreover, the versatility of this design extends its utility across a wide range application, from high-resolution imaging to advanced sensing technologies, the bifocal meta-lens offers precise control over light manipulation and focal points and under varying NA conditions underscores its protentional for transformative applications in optics and photonics.

## Supplementary Information

Supplementary Information.

## Data Availability

The datasets used and/or analyzed during the current study available from the corresponding author on reasonable request.
