# Supplementary material for: Highly Efficient and achromatic mid-infrared silicon nitride meta-lenses
Source: Sci Rep. 2025 Jan 15;15:2008. doi: 10.1038/s41598-024-83728-2 (PMC11736020; doi:10.1038/s41598-024-83728-2)
Supplement: Supplementary file 1 — Supplementary Information. [file 41598_2024_83728_MOESM1_ESM.pdf]

Supplementary information

Attached to

"Mathematical Topology Inverse Design Framework Integrating Layered K-S Aggregation for Achieving High Efficiency, Achromatic Mid-infrared Silicon Nitride Meta-lenses"

Abdullah Maher, Mohamed A. Swillam*

S-1 k-s aggregation parameter adjustment

In case of focusing and reflective meta-lens, the optimization problem depends mainly on the $\boldsymbol{p}$ parameter where it effects directly to the final optimization response as shown from the following equation:

$$\max_{\tilde{\bar{\rho}}} \left(\frac{-1}{p} \ln \left(\sum_{i=1}^{N} e^{-p\left(\Phi_i\left(E_z\left(\lambda_i, r_p, \varepsilon_r\left(\tilde{\bar{\rho}}(r), \lambda_i\right)\right)\right)\right)} \right) \right)$$

The parameter $\boldsymbol{p}$ was systematically varied from 4 to 6. The results indicate that the focusing meta-lens exhibits an approximately stable response at $\boldsymbol{p} \approx \mathbf{5.37}$ (Fig. S1), while the reflective meta-lens achieves stability at $\boldsymbol{p} \approx \mathbf{4.37}$ (Fig. S2).

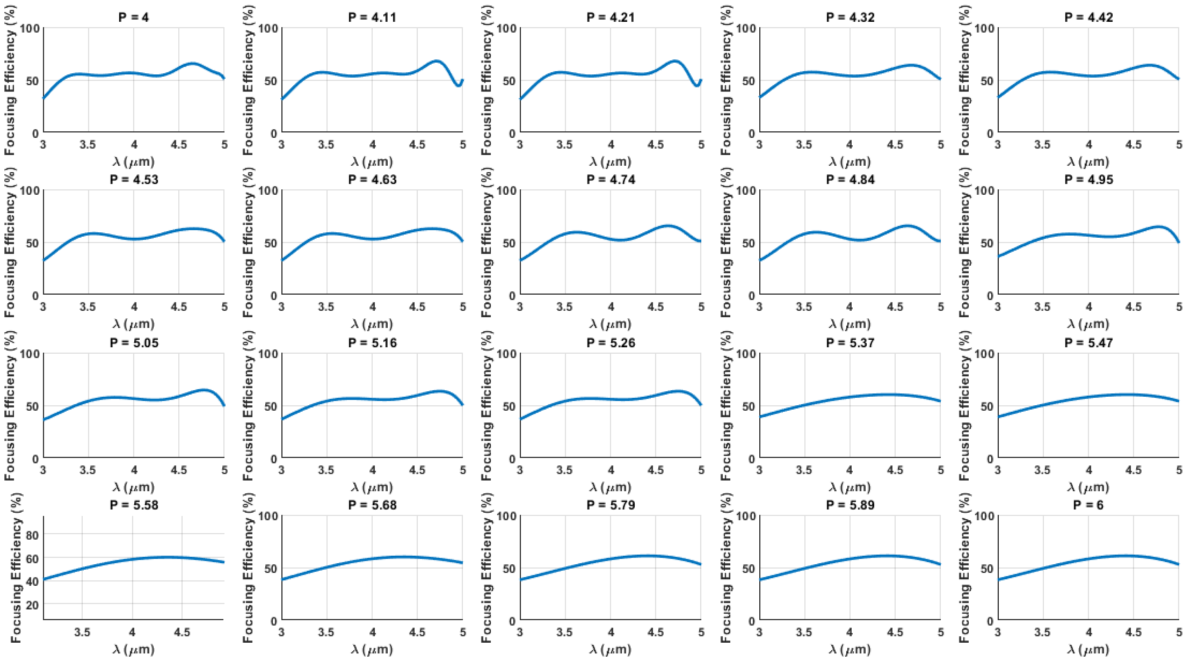

Figure. S1 The focusing efficiency of the focusing meta-lens across the p value.

In case of the bifocal meta-lens, the optimization problem depends mainly on the $\boldsymbol{p_1}$ and $\boldsymbol{p_2}$ parameters where it effects directly to the final optimization response as shown from the following equation:

$$\max_{\tilde{\rho}}\left(-\frac{1}{p_2}\ ln\Sigma_{j=\ 1}^{N_\lambda} e^{\left(\frac{p_2}{p_1} ln\Sigma_{i=1}^{Np}\ e^{-p_1\ I_{i\ ,j}\left(E_z\left(\lambda_j, r_{p_i}, \varepsilon_r\left(\rho(r), \lambda_j\right)\right)\right)}\right)}\right)$$

The parameter $\boldsymbol{p_1}$ was varied from 2 to 4, and $\boldsymbol{p_2}$ was varied from 1 to 3. As shown in Fig. S3, the response achieves stability beginning at $\boldsymbol{p_1} = \boldsymbol{3.16}$ and $\boldsymbol{p_2}\ =\ \boldsymbol{2.16}$.

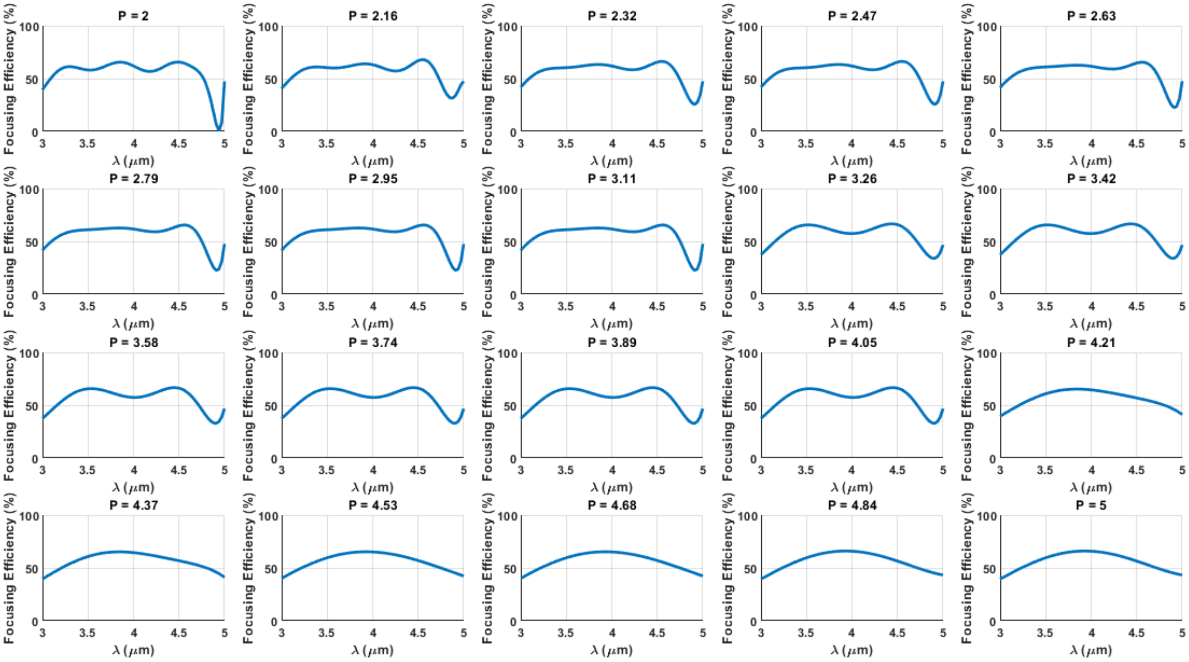

Figure.S2 The focusing efficiency of the reflective meta-lens across the p value.

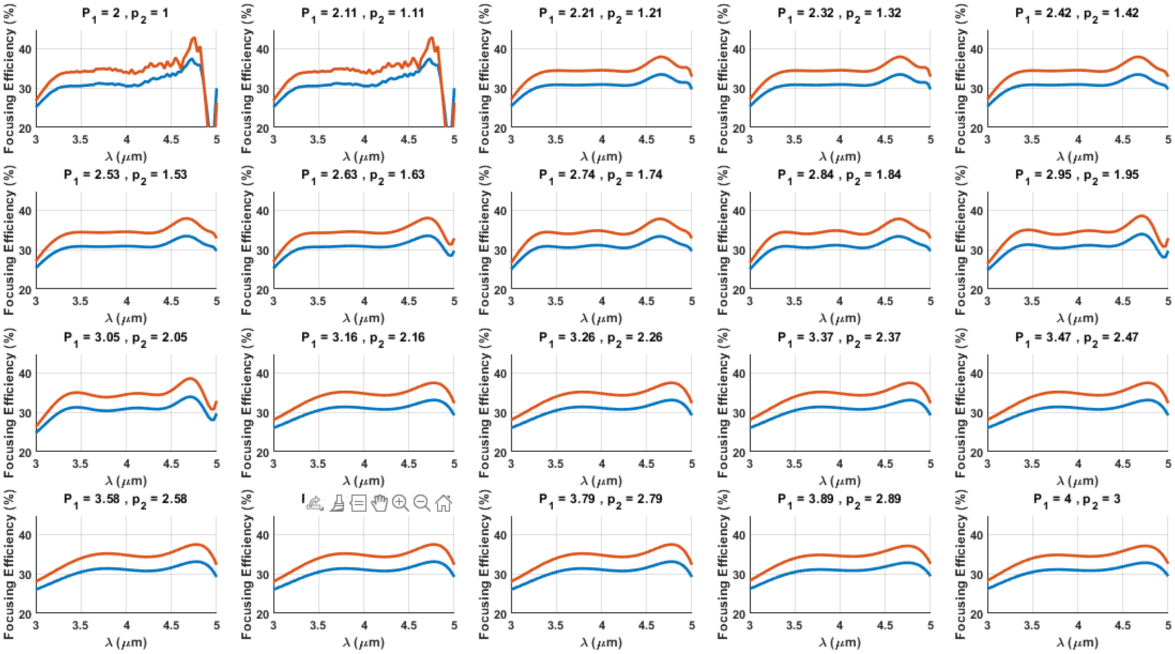

Figure. S3 The focusing efficiency of the bifocal meta-lens across the p value.

S-3 Fmincon function parameters

Algorithm	Interior-point
SpecifyObjectiveGradient	True (user define gradient of the objective function based on FEM)
HessianApproximation	{lbfgs} fmincon calculates the Hessian by a limited-memory, large-scale quasi-Newton approximation.
MaxIterations (focusing meta_lens)	50 (fig. S4(a))
MaxIterations (Reflective meta_lens)	50 (fig. S4(b))
MaxIterations (bifocal meta_lens)	200 (fig. S4(c))

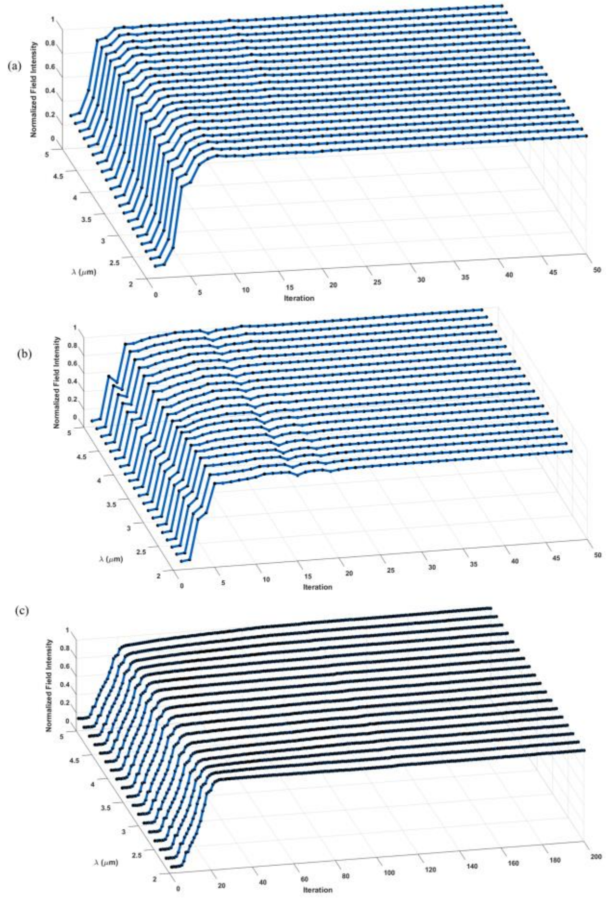

Figure. S4 (a) The convergence history of the focusing meta-lens; (b) The convergence history of the reflective meta-lens; (c) The convergence history of overall aggregation of the bifocal metal-lens.
